# Supramolecular Control of Dual Emission in Macrocycle-Confined Dimers

**DOI:** 10.1021/jacsau.6c00474

**Published:** 2026-05-06

**Authors:** Tianyi Yang, Jacob F. Jones, Song Zhang, Bao Li, Sijia Li, Yizhuo Yu, Yibin Sun, Haichao Liu, Bing Yang, Thomas A. A. Oliver, Guanglu Wu

**Affiliations:** † State Key Laboratory of Supramolecular Structure and Materials, College of Chemistry, 12510 Jilin University, Changchun 130012, P. R. China; ‡ School of Chemistry, 1980 University of Bristol, Cantock’s Close BS8 1TS, U.K.; § State Key Laboratory of Magnetic Resonance and Atomic and Molecular Physics, Innovation Academy for Precision Measurement Science and Technology, Chinese Academy of Sciences, Wuhan 430071, P. R. China

**Keywords:** Dual Emission, Structural Dynamics, Macrocycle, Noncovalent Dimer, Supramolecular System

## Abstract

Control over luminescent properties is conventionally achieved by designing rigid, static packing geometries. Yet, chromophores within these assemblies naturally undergo continuous relative motion; harnessing this often-overlooked dynamic flexibility to actively dictate excited-state outcomes offers a powerful new dimension in materials design. Here, we introduce a supramolecular strategy to systematically control dual emission by restricting the structural dynamics of macrocycle-confined dimers. Utilizing cucurbit[8]­uril (CB[8]) macrocyclic host and bis­(phenylpyridinium) (BPP) guests, we construct precise 2:1 and 2:2 host–guest complexes to establish dynamic and static mobility limits within a unified framework. Cavity-confined dimerization induces a unique intrinsic dual emission. By progressively tightening structural restriction—moving from the fluxional 2:1 complex to the clamped 2:2 architecture, and further to a rigidly sodium-bridged framework—the dominant emission cleanly shifts from a short-wavelength state to a long-wavelength state, accompanied by a dramatically enhanced fluorescence quantum yield. Time-resolved spectroscopy reveals that this supramolecular confinement actively governs the kinetics of excited-state relaxation, definitively linking motional freedom to the resulting functional photoluminescence. Collectively, these results showcase the controlled restriction of supramolecular dynamics as an innovative, general design principle for tailoring programmable optoelectronic materials.

## Graphical abstract

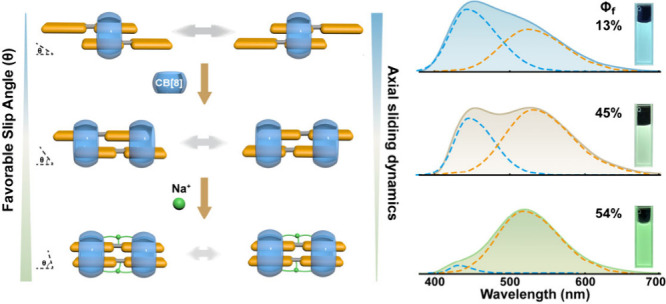

## Introduction

Interactions among adjacent chromophores orchestrate the photophysical behavior that ultimately determines the performance of luminescent and optoelectronic materials.^1–4^ In most molecular and materials systems, this behavior is conventionally tailored by designing rigid, static structural factors, such as interchromophore spacing,^5–7^ slip angle,^8,9^ and specific stacking geometry.^10–12^ Subtle variations in such packing configurations are well recognized for their ability to reshape excitonic coupling and critically modulate emissive properties.^4,5,13–15^

Implicit in this design framework is the assumption that chromophore assemblies are structurally fixed on the time scale of excited-state evolution. However, at the molecular scale, chromophore assemblies are inherently dynamic, continuously sampling multiple geometries. Internal motions such as relative sliding,^16,17^ axial displacement,^18^ and local reorganization^19–21^ frequently occur on time scales that parallel excited-state processes. These intrinsic structural flexibilities can actively redirect emissive pathways,^22–24^ rather than merely acting as passive perturbations to static packing. Yet, despite extensive studies on packing-dependent photophysics,^3,4,12,25–28^ the deliberate harnessing of interchromophore motion itself to actively govern and program luminescent outcomes remains a largely unexplored frontier.

Supramolecular host–guest systems provide an ideal platform to realize this dynamic control. In particular, cucurbit[8]­uril (CB[8]) can confine two chromophores within a well-defined cavity,^29,30^ enforcing strong excitonic coupling while crucially retaining tunable degrees of relative motion.^31–33^ Distinct binding motifs, including 2:1,^34–37^ 2:2,^38–48^ and 1:1 self-folded^38,39,49–53^ complexes offer access to diverse regimes of structural restriction without altering the intrinsic electronic building blocks. Such systems therefore present a unique opportunity to construct precisely defined dynamic and static limits within a unified supramolecular framework, allowing us to directly correlate motional freedom with versatile emissive features.

Here we exploit CB[8]-confined bis­(phenylpyridinium) dimers to systematically modulate interchromophore motion and translate this supramolecular control into tunable dual emission. Taking advantage of the distinct intrinsic mobilities of 2:1 and 2:2 host–guest complexes (Scheme 1), we demonstrate that the degree of motional freedom—ranging from fluxional sliding to clamped, rigid stacking—directly governs the accessible emissive states, peak emission wavelengths, and fluorescence quantum yields. By further enforcing rigidity through cation-bridged confinement, we reveal how suppressing structural dynamics can act as a precise dial for selectively channelling emission. Collectively, these results showcase the controlled restriction of supramolecular dynamics as an innovative and general design principle, elevating motional freedom from a passive molecular trait to an active, programmable parameter for advanced luminescent materials.

**Scheme 1:**
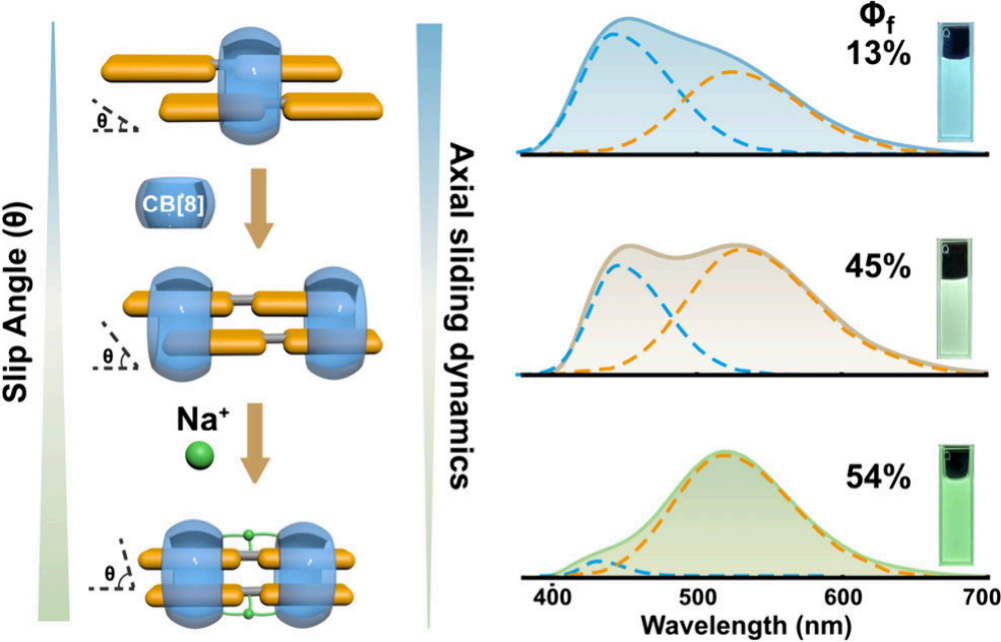
Increasing Structural Confinement, from Freely Sliding to Strongly Constrained Noncovalent Dimers, Tunes the Balance between Short- and Long-Wavelength Emission in Macrocycle-Confined Assemblies

## Results and Discussion

### Distinct Dynamics in 2:1 and 2:2 Complexes

In this study, we selected bis­(phenylpyridinium) (BPP) as a model guest to construct CB[8]-confined noncovalent dimers with well-defined binding motifs (Figure 1). BPP is a simple, symmetric molecule with a conjugated and cationic structure that makes it photophysically active in the near-ultraviolet and ensures strong binding to the CB[8] cavity. Importantly, our recent work^54^ has shown that BPP engages with CB[8] in stoichiometrically precise 2:1 and 2:2 complexes, which exhibit distinct intracomplex dynamic regimes, thereby providing an ideal platform to probe how differences in structural mobility translate into variation in excited-state behavior. The formation of these complexes is confirmed by ^1^H NMR spectroscopy (Figure 1). Because the carbonyl portals of CB[8] generate a ring-current shielding effect, the extent of changes in proton chemical shift directly reflects the guest’s relative position within the host: larger upfield shifts indicate proximity to the cavity center, whereas smaller shifts correspond to more peripheral locations.^55^

**Figure 1:**
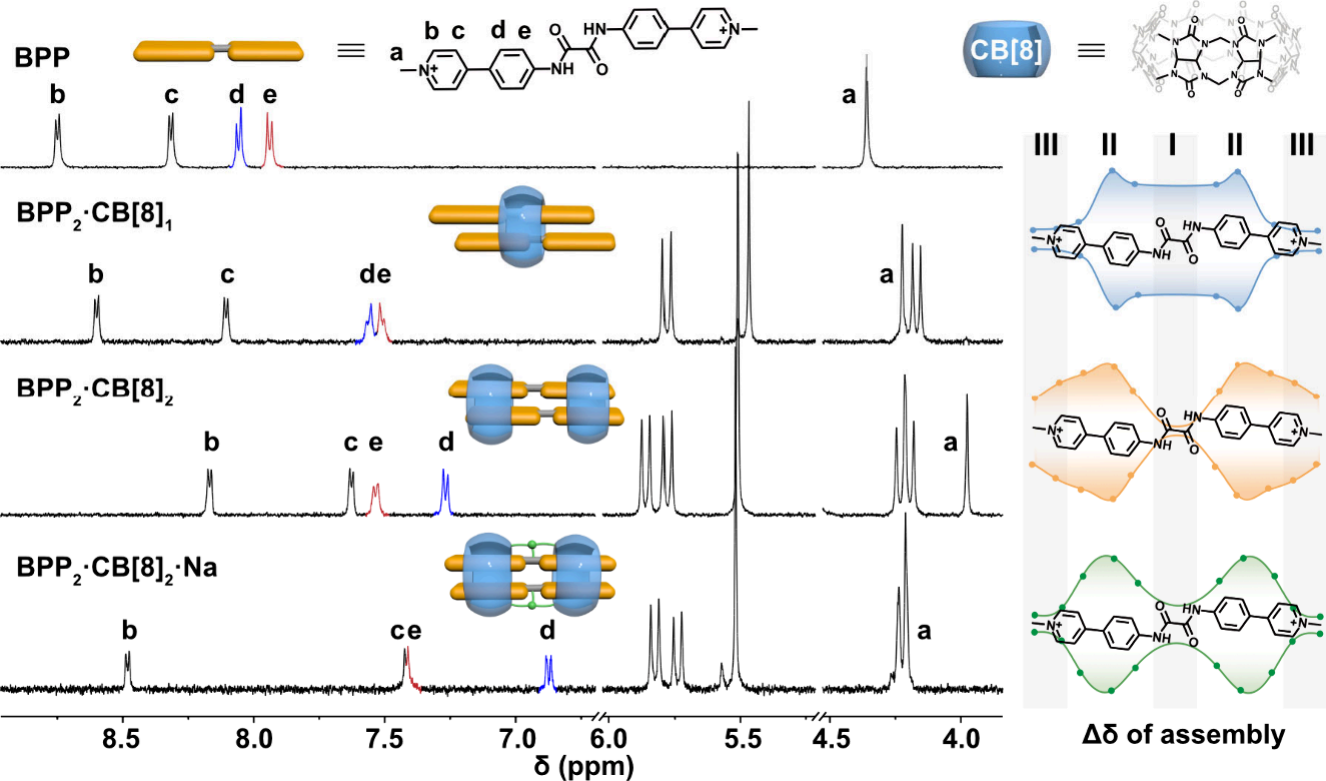
^1^H NMR spectra (500 MHz, D_2_O, 298 K) of BPP and its CB[8] complexes: 2:1 (BPP_2_·CB­[8]_1_), 2:2 (BPP_2_·CB­[8]_2_), and Na^+^-bridged BPP_2_·CB­[8]_2_ (BPP_2_·CB­[8]_2_·Na). Right: Δδ profiles (relative to free BPP) reconstructed from assigned proton shifts (see Figure S6) illustrate the average CB[8] location along the BPP axis (regions I–III): in BPP_2_·CB­[8]_1_ (blue), upfield shifts extend across the central region, consistent with rapid axial sliding; in BPP_2_·CB­[8]_2_ (orange), shifts localize at the termini, indicating end-residing macrocycles; upon Na^+^ addition (green), the Δδ maximum moves toward the center, consistent with cation-bridged contraction of the two CB[8] rings. All samples were reported at a CB[8] concentration of approximately 80 μM.

In the 2:1 complex (BPP_2_·CB­[8]_1_), protons across the central regions (I and II in Figure 1) of BPP display pronounced upfield shifts, consistent with rapid motion of CB[8] along the guest axis on the NMR time scale (microsecond to millisecond time scales). Such mobility not only allows the guests to sample different positions within the host macrocycles but also, under weak confinement, permits relative sliding between the two BPP units, yielding variable slip angles. The combination of host sliding and guest flexibility renders BPP_2_·CB­[8]_1_ a relatively dynamic assembly. The relatively well-resolved NMR resonances suggest that, although BPP_2_·CB­[8]_1_ is more dynamic overall, it does not undergo pronounced fast exchange under the present conditions.

In contrast, in the 2:2 complex (BPP_2_·CB­[8]_2_), upfield shifts are localized mainly at the terminal regions (II and III), indicating that two CB[8] macrocycles preferentially reside at the termini of the dimer. This multivalent binding pattern effectively clamps the two BPP units, restricting axial displacement and suppressing the relative slip between the two stacked chromophores. As a result, BPP_2_·CB­[8]_2_ adopts a more static configuration, compared to BPP_2_·CB­[8]_1_, where conformational mobility is substantially reduced. Consistently, the resolved splitting of the CB[8] methylene signals supports that BPP_2_·CB­[8]_2_ is kinetically stable on the NMR time scale.

Thus, BPP_2_·CB­[8]_1_ and BPP_2_·CB­[8]_2_ represent two opposing (dynamic and static) limits of supramolecular mobility within the same host–guest framework. This distinction is further supported by DFT calculations (Figure S29), which show a much larger energy separation between representative slipped conformations of BPP_2_·CB­[8]_2_ than of BPP_2_·CB­[8]_1_, consistent with a more rigid conformational preference for the 2:2 complex and a shallower conformational landscape for the 2:1 complex. This contrast provides a clear basis for investigating how differences in motional freedom translate into distinct fluorescence maxima and excited-state relaxation pathways, as revealed below.

### Dual Emission Enabled by Cavity-Confined Dimerization

The steady-state spectra reveal that dual emission emerges as an intrinsic feature of BPP dimers formed within the CB[8] cavities. Compared to free BPP, encapsulation by CB[8] produces broadened and red-shifted absorption bands (Figure 2a), indicative of strong excitonic coupling between cofacially stacked chromophores. Moreover, BPP_2_·CB­[8]_1_ and BPP_2_·CB­[8]_2_ display distinct absorption maxima, reflecting subtle differences in their stacking arrangement.

**Figure 2:**
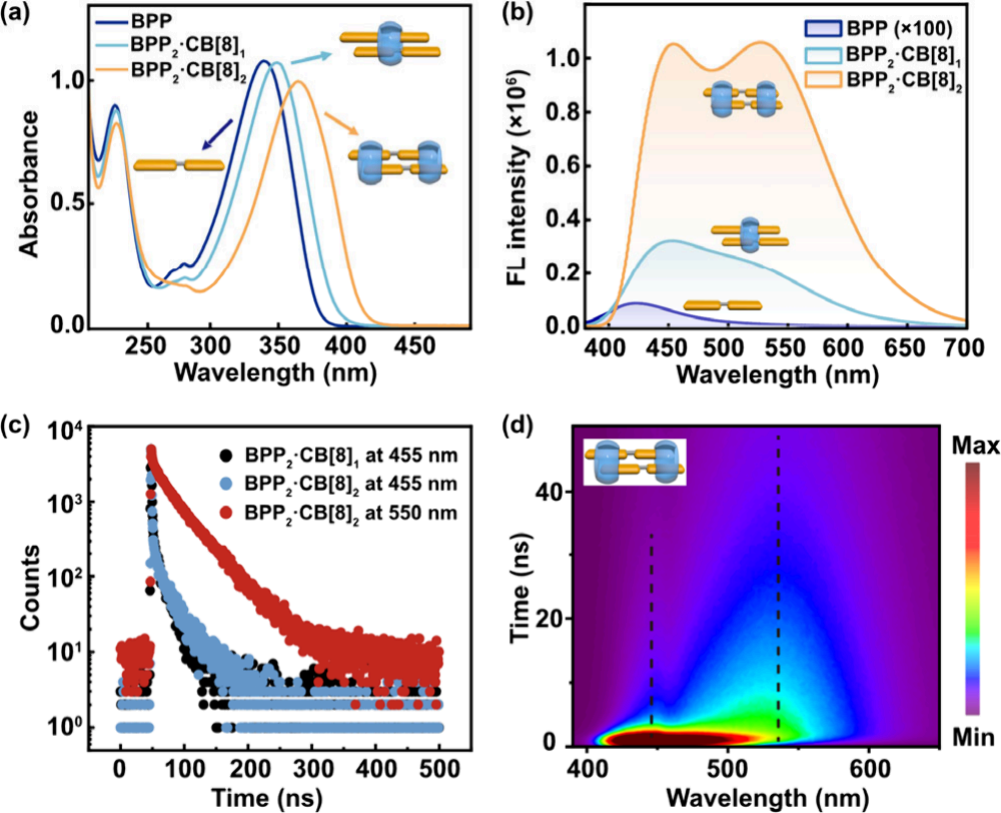
Steady-state and time-resolved photophysical characterization of CB[8]-confined BPP dimers in water. (a) Steady-state absorption and (b) emission spectra of BPP (blue), BPP_2_·CB­[8]_1_ (cyan), and BPP_2_·CB­[8]_2_ (orange). (c) Time-dependent fluorescence decay of BPP_2_·CB­[8]_1_ at 455 nm (black) and BPP_2_·CB­[8]_2_ at 455 nm (blue) and 550 nm (red). (d) Time-resolved fluorescence spectra of BPP_2_·CB­[8]_2_. All samples were measured at a uniform BPP concentration of 20 μM at 298 K.

Free BPP is limited to a weak blue emission at 423 nm (Φ_f_ ≈ 4%, Table 1), but upon encapsulation by CB[8], both complexes exhibit broad dual emission (Figure 2b), confirming that formation of a noncovalent dimer within the cavity is essential for generating multiple emissive states. The two assemblies nevertheless differ in their emission profiles: BPP_2_·CB­[8]_1_ shows a dominant short-wavelength band (∼450 nm), whereas BPP_2_·CB­[8]_2_ exhibits a more intense long-wavelength band (∼527 nm). The following control experiments rule out the possibility that these observations arise from a ground-state equilibrium between distinct species with different corresponding electronic structures. Increasing the CB[8] ratio up to three equivalents or varying the complex concentration by nearly 2 orders of magnitude produced negligible changes in the relative intensities of the two emission bands (Figure S15). These results demonstrate that the dual-emission behavior originates from intrinsic dynamic excited-state processes within the confined dimers,^56^ rather than from the coexistence of multiple ground state species.

**Table 1:** Photophysical Parameters of BPP and Its CB[8]-Mediated Complexes^a^

Species	λ_abs_ (nm)	λ_em_ (nm)	τ_f_ (ns, [relative amplitude])	Φ_f_ (%)
BPP	340	430	<1.4 (91.1%), 7.5 ± 0.3 (8.9%)	4
BPP_2_·CB[8]_1_	350	455	<1.4 (58.5%), 18.4 ± 0.3 (41.5%)	13
BPP_2_·CB[8]_2_	363	445	<1.4 (52.3%), 38.0 ± 0.2 (47.7%)	45
		550	<1.4 (9.7%), 41.6 ± 0.2 (90.3%)	
BPP_2_·CB[8]_2_·Na	358	520	41.5 ± 0.2	54

a Aqueous solution at 298 K with [BPP] = 20 μM. For BPP_2_·CB­[8]_2_·Na, the NaBr concentration was 300 equiv. relative to the complex.

Time-resolved measurements provide direct evidence for distinct emissive species in both assemblies. Consistent with their closely spaced dual-emission bands, BPP_2_·CB­[8]_1_ and BPP_2_·CB­[8]_2_ both exhibit biexponential fluorescence decays (Figure 2c). At 455 nm, the two complexes share nearly identical short-lived decay components that decay faster than our instrument response (τ < 1.4 ns) (Table 1), indicating a common nonradiative decay pathway for the short-wavelength channel. The key distinction emerges at the long-wavelength channel: in BPP_2_·CB­[8]_2_, the 550 nm emission is dominated by a long-lived species (τ ≈ 40 ns), whereas in BPP_2_·CB­[8]_1_ the corresponding species shows a shorter lifetime of τ ≈ 18 ns. This difference highlights the stronger structural constraint in BPP_2_·CB­[8]_2_, which stabilizes the red-shifted emissive state relative to the more dynamic BPP_2_·CB­[8]_1_. Correspondingly, the fluorescence quantum yield rises from ∼ 13% in BPP_2_·CB­[8]_1_ to ∼ 45% in BPP_2_·CB­[8]_2_, consistent with suppression of nonradiative decay in the more rigid assembly.

Time-resolved fluorescence spectra of BPP_2_·CB­[8]_2_ further clarify the sequential nature of the process (Figure 2d). Following excitation, a short-wavelength band appears promptly and is eventually replaced by a long-wavelength emission feature, suggesting that the latter originates from the population initially residing in the short-wavelength emitting state. Both complexes access the same emissive states (Figures S16–S17), but their relative populations differ, with BPP_2_·CB­[8]_1_ biased toward the short-lived blue-shifted species and BPP_2_·CB­[8]_2_ favoring the long-lived red-shifted species.

### Excited-State Kinetics Revealed by Transient Absorption

Femtosecond transient absorption (TA) spectroscopy provides direct insight into the excited-state evolution of the BPP–CB[8] complexes (Figure 3, Figures S21–S23). Both BPP_2_·CB­[8]_1_ and BPP_2_·CB­[8]_2_ exhibit a sequential evolution involving three distinct species. Immediately after excitation, a short-wavelength stimulated emission (SE) band appears near 450 nm (species A), directly corresponding to the short-wavelength fluorescence observed in steady-state spectra. This emissive state then decays into a nonemissive intermediate (species B), characterized by the absence of any SE, and thus ascribed to an optically dark state. Subsequently, a second SE band emerges at ∼ 530 nm (species C), matching the longer-wavelength fluorescence band. This “bright–dark–bright” sequence unambiguously establishes that the two emission bands originate from distinct emissive states connected through a sequential relaxation via an intermediate dark state, and are entirely consistent with the time-resolved fluorescence results.

**Figure 3:**
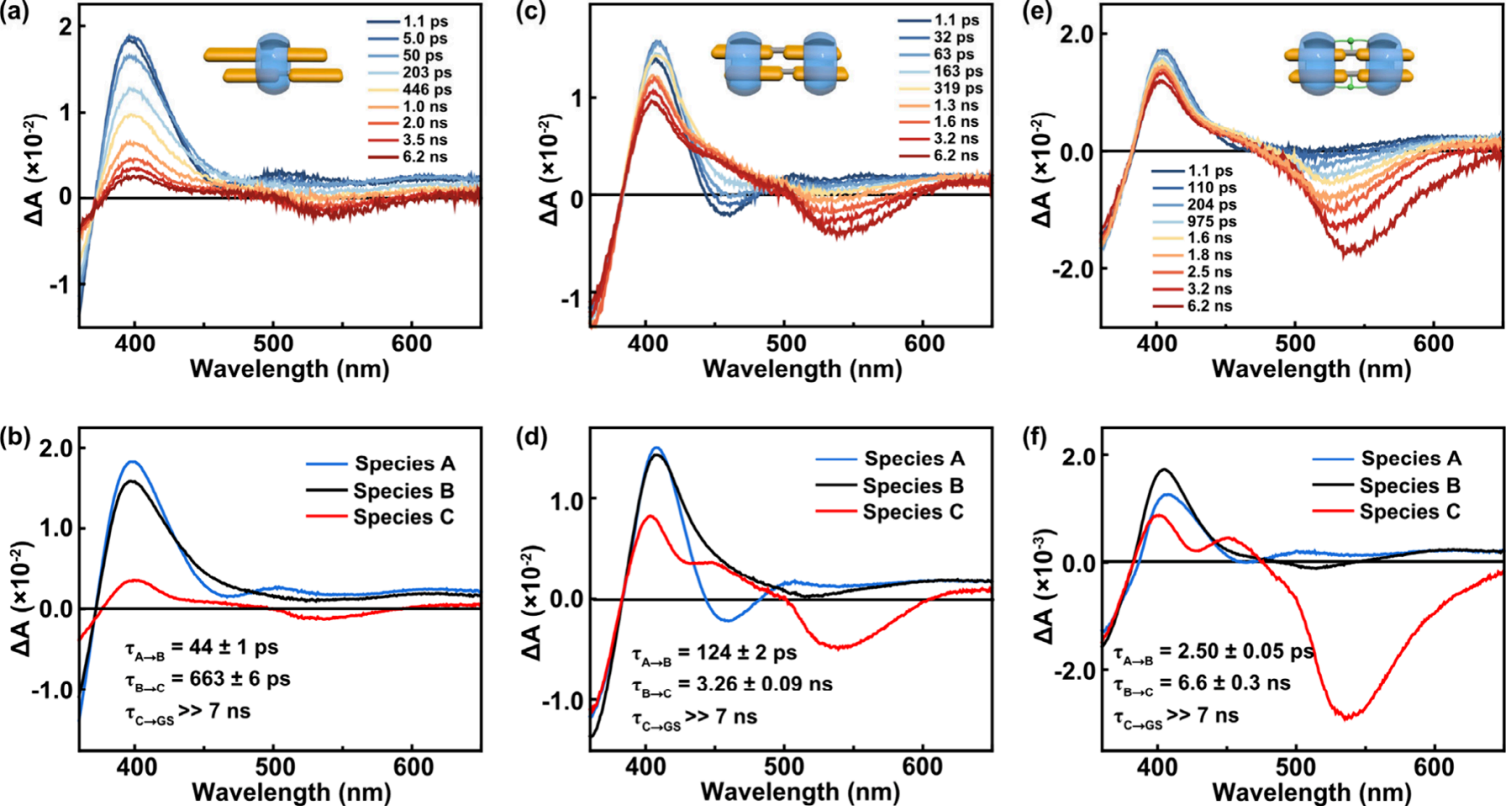
Femtosecond transient absorption (TA) spectra of (a,b) BPP_2_·CB­[8]_1_, (c,d) BPP_2_·CB­[8]_2_, and (e,f) BPP_2_·CB­[8]_2_·Na (300 equiv. NaBr) in H_2_O at 298 K following excitation at 350 nm (1 μJ per pulse). Panels (a), (c), and (e) show the time-resolved ΔA spectra, while (b), (d), and (f) present the corresponding species-associated spectra (SAS) obtained by global fitting using a sequential kinetic model (A → B → C → GS). All samples were measured at a uniform BPP concentration of 40 μM at 298 K.

Kinetic analysis further highlights the effect of intracomplex dynamics. In BPP_2_·CB­[8]_1_, the A→B conversion occurs within tens of picoseconds (τ ≈ 44 ps), followed by a relatively fast B→C transition (τ ≈ 663 ps) (Figure 3a). By contrast, in BPP_2_·CB­[8]_2_ these processes are markedly slower: the A→B step extends to ∼ 124 ps, while the subsequent B→C conversion is prolonged to several nanoseconds (τ ≈ 3.3 ns) (Figure 3b). Within a qualitative energy landscape picture, increased structural restriction narrows the accessible coordinate space and elevates transition barriers to reorganization, thereby slowing state-to-state interconversion. The presence of the second CB[8] macrocycle thus locks the dimer into a more rigid geometry, suppressing relative sliding and leading to slower excited-state relaxation.

### BPP_2_·CB­[8]_2_ Further Constrained by Cation Bridging

The portals of CB[8] are lined with carbonyl groups that can readily coordinate or interact with metal cations.^18,43^ We therefore speculated that suitable cations might bridge two adjacent CB[8] macrocycles in BPP_2_·CB­[8]_2_, providing an additional level of structural confinement beyond that imposed by the two macrocycles themselves. Such ion-mediated bridging would be expected to further suppress intracomplex motion and enhance the rigidity of the stacked dimer.

Steady-state emission directly reflects this effect. Upon gradual addition of Na^+^, the short-wavelength band at ∼ 450 nm progressively diminishes, while the long-wavelength band at ∼ 530 nm intensifies, and dominates at high ion concentrations (Figure 4a). This trend indicates that rigidification from ion bridging selectively stabilizes the long-wavelength emissive state, consistent with the markedly prolonged fluorescence lifetime of 41.5 ns observed for the Na^+^-bridged complex (Table 1, Figure S18). The effect is pronounced for Na^+^ and K^+^ but negligible for other cations (Figure 4b), likely reflecting their optimal ionic radii and stronger affinity for the CB[8] carbonyl portals, in contrast to ions too large or too small to bridge effectively.^43^ This specificity is further supported by our observation that the emission response remains unchanged despite variations in the accompanying anion (e.g., Cl^–^, Br^–^, I^–^, CO_3_^2–^, HCO_3_^–^, SO_4_^2–^, or HSO_4_^–^) at a constant Na^+^ concentration, even when the ionic strength fluctuates significantly. Furthermore, other cations (e.g., Li^+^, NH_4_^+^, Cs^+^) fail to induce a similar dramatic spectral transition, even when the ionic strength is comparable to that of NaCl, which demonstrates that the observed response is driven by cation-specific bridging rather than a general salt effect.

**Figure 4:**
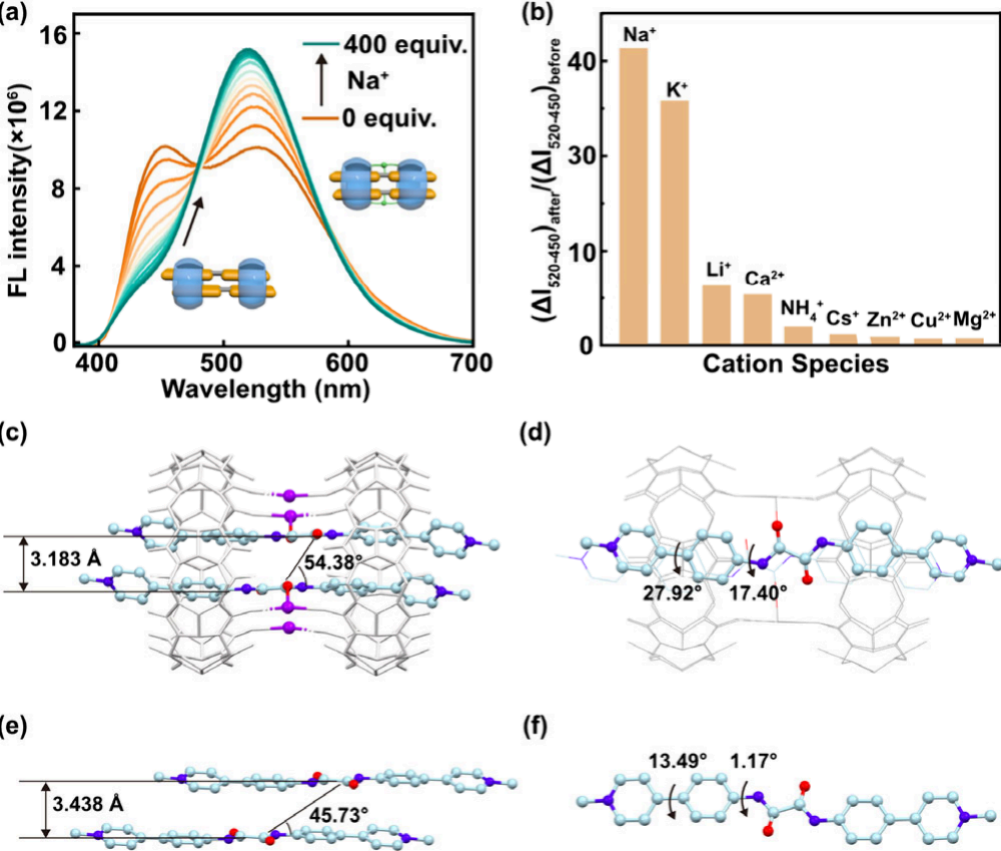
(a) Emission spectra of BPP_2_·CB­[8]_2_ upon addition of increasing equivalents of NaBr in aqueous solution. (b) Cation-dependent emission response measured with 300 equiv of metal ions, showing pronounced selectivity for Na^+^ and K^+^ (all chloride salts, [BPP] = 40 μM). (c,d) Crystal structure of BPP_2_·CB­[8]_2_·Na, showing Na^+^-bridged portals between adjacent CB[8] macrocycles. (e,f) Crystal structure of free BPP for comparison. Interchromophore spacing, slip angle, and intramolecular torsion angle are labeled. (CB[8] shown in gray, C in cyan, N in blue, O in red, Na in purple; H atoms, solvent molecules, and counterions omitted for clarity).

NMR spectra provide complementary evidence. Incremental addition of Na^+^ induces systematic chemical shift changes (Figure 1): terminal protons (region III) shift downfield, while central protons (regions I and II) move upfield, consistent with stronger shielding as the two CB[8] rings are drawn toward the center. The titration profile (Figures S7–S8) shows a two-stage evolution, suggesting an initial Na^+^-induced contraction of the CB[8] separation, followed by additional anchoring through interactions with the amide carbonyls of BPP. Single-crystal analysis corroborates this picture (Figure 4c–f), revealing Na^+^ ions bridging adjacent CB[8] portals and additionally interacting with the amide carbonyls of BPP, shortening the interchromophore distance (3.18 Å vs 3.44 Å in free BPP) and imposing torsional distortion on the BPP backbone. The Na^+^-bridged complex (BPP_2_·CB­[8]_2_·Na) curiously also exhibits a blue-shift in its absorption maximum relative to the unbridged BPP_2_·CB­[8]_2_ (Figure S19), indicative of enhanced H-type aggregation^11,57^ consistent with the large slip angle of 54.38° observed in the crystal structure (Figure 4c). Spectroscopic and structural data thus converge to show that Na^+^ bridging enforces a more rigid, ion-locked architecture, further suppressing intracomplex motion beyond the confinement imposed by two CB[8] macrocycles alone.

### A Qualitative Energy Landscape Framework

Transient absorption experiments highlight the kinetic consequences of the added restriction by ion bridging (Figure 3e,f, Figures S24–S27): the assemblies still follow the characteristic “bright-dark-bright” sequence, but the time scales for interconversion are significantly longer due to the greater rigidity. The B→C conversion slows from ∼ 3.3 ns in the unbridged complex to 6.6 ns under 300 equiv of Na^+^ (Figure 3f) and to nearly 8 ns under 500 equiv of Na^+^ (Figure 5a), reflecting a higher barrier for accessing the long-wavelength state. In contrast, the A→B step becomes extremely fast, approaching the instrument response limit. We attribute this to a geometric bias introduced by ion bridging: the ion-locked ground-state geometry closely resembles that of Species B, placing the system near the downhill side of the A–B barrier (Figure 5b, green pathway) immediately upon excitation. As a result, the population rapidly relaxes into B with little observable residence in A, as reflected in the negligible short-wavelength SE at early delay times (Figure 3e,f).

**Figure 5:**
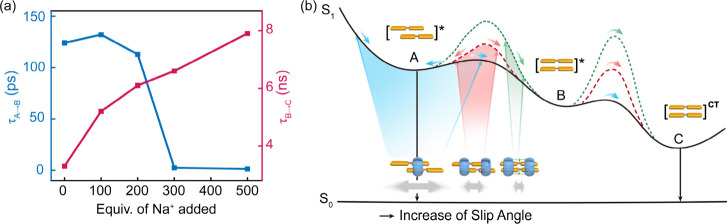
(a) Evolution of kinetic lifetimes τ_A→B_ and τ_B→C_ of BPP_2_·CB­[8]_2_ (40 μM based on BPP units) upon addition of increasing equivalents of NaBr (0–500 equiv); (b) schematic energy landscape illustrating the sequential excited-state relaxation (A → B → C) in CB[8]-confined dimers.

Although the underlying mechanism is likely more complex, the observed kinetic differences can be rationalized within a simplified energy landscape framework (Figure 5b). This framework also explains the contrasting behaviors of BPP_2_·CB­[8]_1_ and BPP_2_·CB­[8]_2_ (Figure 5b, blue and red), and is inspired by recent theoretical work by Krieger et al.^57^ who examined the excitonic interactions of aggregates. The multiple excited state minima and their dependence on slip angle arise from switching between J- (bright) and H- (dark) type aggregate minima, as the alignment and signs of the two monomer transition density lobes change. For instance, A→B→C interconversion corresponds to excited state transfer between J-, H- and ultimately a lower-energy J-type aggregate minima. In the more dynamic BPP_2_·CB­[8]_1_, sliding freedom biases the initial geometry toward the uphill side of the A–B barrier, leaving a larger fraction of the population trapped in A and thus producing stronger short-wavelength SE. By contrast, BPP_2_·CB­[8]_2_, with reduced mobility, distributes more evenly across both sides of the barrier, enabling partial but slower conversion into B and subsequently into C. Ion bridging further shifts this balance by locking the geometry close to B, eliminating the residence in A and funnelling the population directly into the long-wavelength pathway.

## Conclusions

In summary, this work demonstrates that programmable control over luminescent properties can be elegantly achieved by harnessing the inherent dynamics of supramolecular assemblies. Using CB[8]-confined BPP dimers as a versatile model platform, we show that simply tuning the degree of stacking mobility determines the distribution of accessible emissive states, resulting in distinct, tunable dual-emission signatures. By imposing further structural restriction through cation bridging, we can almost exclusively bias relaxation toward the long-wavelength fluorescence pathway, vividly illustrating how hierarchical confinement directly translates into systematic control over emission color and quantum efficiency.

Crucially, these findings establish the controlled restriction of intercomponent dynamics as a powerful and highly general design principle for optoelectronic materials. While conventional strategies for emission control predominantly rely on rigid covalent architectures or static crystal packing motifs, our study demonstrates that the degree of motional freedom itself—such as relative sliding, reorganization, and their deliberate restriction—can serve as a robust lever to program emissive outcomes. This perspective suggests that rather than attempting to eliminate molecular dynamics, actively engineering their structural boundaries can unlock powerful new routes to tailor programmable photoluminescent materials, exciton lifetimes, and energy-transfer systems.

## Supplementary Material



## Notes

Additional experimental details, including materials and methods, and additional data, including ^1^H NMR spectra, ^13^C NMR spectra, COSY spectra, DOSY spectra, NOESY spectra, LC-MS, ITC, TA spectra, UV–vis absorption spectra, fluorescence emission spectra, TCSPC, and single-crystal results (PDF)
