# Supplementary material for: Supramolecular Control of Dual Emission in Macrocycle-Confined Dimers
Source: JACS Au. 2026 May 6;6(5):3059–66. doi: 10.1021/jacsau.6c00474 (PMC13213490; doi:10.1021/jacsau.6c00474)
Supplement: Supplementary file 1 [file au6c00474_si_001.pdf]

Supporting Information

Supramolecular Control of Dual Emission in Macrocycle-Confined Dimers

Tianyi Yang[1], Jacob F. Jones[2], Song Zhang[3], Bao Li[1], Sijia Li[1], Yizhuo Yu[1], Yibin Sun[1], Haichao Liu[1], Bing Yang[1], Thomas A. A. Oliver[2*], Guanglu Wu[1*]

[1]State Key Laboratory of Supramolecular Structure and Materials, College of Chemistry, Jilin University, Changchun 130012, P. R. China

[2]School of Chemistry, University of Bristol, Cantock's Close, BS8 1TS, U.K.

[3]State Key Laboratory of Magnetic Resonance and Atomic and Molecular Physics, Innovation Academy for Precision Measurement Science and Technology, Chinese Academy of Sciences, Wuhan 430071, P. R. China

[*]Email: tom.oliver@bristol.ac.uk; guanglu@jlu.edu.cn

Table of Contents:

1. Materials and methods 2

2. Synthesis and characterization of arylpyridinium derivatives 4

3. NMR Spectra of BPP and its CB[8]-mediated complexes....................7

4. Photophysical study on BPP and its CB[8]-mediated complexes........13

5. Transient absorption of BPP and its CB[8]-mediated complexes........16

6. Single crystal results 23

7. Geometry optimization...26

8. References 27

1. Materials and methods

Materials. All reagents and solvents as follows were purchased from commercial sources and used without further purification: 4-(pyridin-4-yl)aniline (99%, Adamas), oxalyl chloride (99%, Adamas), and methyl iodide (99%, Sigma-Aldrich). Unless otherwise noted, all metal salts were used in their chloride form. Cucurbit[8]uril (CB[8]) was synthesized according to the published procedure.[1]

Isothermal Titration Calorimetry (ITC). All ITC experiments were conducted using a TA ITC Auto at 298 K in deionized water. In each experiment, the host molecule (CB[8]) was placed in the sample cell, and the guest molecule was loaded in the injection syringe. The concentration of CB[8] was determined by titration with a standard solution of 1-Adamantanamine hydrochloride (ADA). The titration traces were integrated using NITPIC, fitted with SEDPHAT, and visualized with GUSSI.

Nuclear Magnetic Resonance Spectroscopy (NMR). ^{1}H NMR, ^{1}H-^{1}H COSY, ^{1}H-^{1}H NOESY, and DOSY spectra were acquired at 298 K using a Bruker AVANCE III 500 MHz spectrometer, Bruker AVANCE III 600MHz spectrometer, and a QOne AS 400MHz spectrometer.

DOSY experiments were carried out using a modified version of the Bruker sequence ledbpgppr2s typically involving 8 scans over 16 steps of gradient variation from 10% to 80% of the maximum gradient. Diffusion coefficients were evaluated in Dynamic Centre (a standard Bruker software) by fitting the intensity decays according to the following Equation:

$$I = I_0 e^{-D\gamma^2 g^2 \delta^2 (\Delta - \delta/3)}$$

where I and Io represent the signal intensities in the presence and absence of gradient pulses, respectively. D is the diffusion coefficient, γ= 4257.7 Hz/G is the ^{1}H gyromagnetic ratio, δ= 2.8 ms is the duration of the gradient pulse, Δ= 70 ms is the total diffusion time and g is the applied gradient strength. The Monte Carlo simulation method was used for the error estimation of fitting parameters with a confidence level of 95%.

Liquid Chromatography-Mass Spectrometry (LC-MS). The mass spectrometry was performed using an Agilent1290-Bruker micrOTOF QII.

UV/Vis Spectroscopy. UV/Vis steady-state absorption spectra were recorded using a Shimadzu UV-2600i spectrophotometer at 298 K. Unless otherwise specified, the optical path length was 1 cm.

Single-Crystal X-ray Diffractions. X-ray diffraction data were collected using synchrotron radiation and MAR325 CCD detector at Shanghai Synchrotron Radiation BL17B Beamline.

Time-Correlated Single Photon Counting (TCSPC). TCSPC was recorded at FLS 920 Spectrofluorometer, Edinburgh Instruments with a laser of 350 nm for the fluorescence lifetime measurements. The instrument response was 1.4 ns full width half max (FWHM). Time-resolved Fluorescence Spectroscopy was conducted via the TCSPC technique. Fluorescence decay curves were collected across the emission range of 390-700 nm at 2 nm intervals. These wavelength-dependent decay profiles were then assembled to construct a three-dimensional spectro-temporal map, depicting fluorescence intensity as a function of both emission wavelength and time.

Fluorescence Quantum Yield Measurements. Absolute fluorescence quantum yield was also recorded at FLS920 Spectrofluorometer, Edinburgh Instruments with an integrating sphere module (SC-30).

Femtosecond Transient Absorption (TA) Spectroscopy. Femtosecond transient absorption spectra were recorded on a HELIOS Fire spectrometer (Ultrafast Systems LLC) (probe wavelength range: 320-640 nm). Solution samples were prepared in a 2 mm path length quartz cuvette. All the samples were dissolved in H_2O excited at 350 nm (1μJ/pulse) at 298K ambient condition.

2. Synthesis and characterization of arylpyridinium derivatives

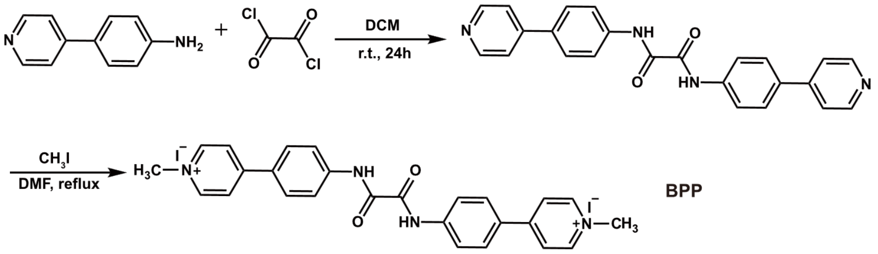

Scheme S1. Synthetic route of **BPP**.

N^1,N^2-bis(4-(pyridin-4-yl)phenyl)oxalamide. 4-(Pyridin-4-yl)aniline (0.17 g, 1 mmol) was dissolved in anhydrous dichloromethane (40 mL). The solution was cooled in an ice-water bath, and oxalyl chloride (33.9 μL, 0.40 mmol) was added dropwise. The reaction mixture was stirred at room temperature for 24 hours. After completion of the reaction, the precipitate was collected by filtration, washed with dichloromethane (20 mL), and dried under vacuum, yielding a pink solid N^1,N^2-bis(4-(pyridin-4-yl)phenyl)oxalamide (0.11 g, 70%). ^{1}H NMR (500 MHz, DMSO-d_6) δ 11.09 (s, 2H), 8.63 (d, J = 6.1 Hz, 4H), 8.05 (d, J = 8.8 Hz, 4H), 7.88 (d, J = 8.7 Hz, 4H), 7.74 (d, J = 6.3 Hz, 4H). LC-MS *(m/z)*: calculated for $C_{24}H_{19}N_4O_2^+$ ([M + H]$^+$): 395.2; found: 395.2.

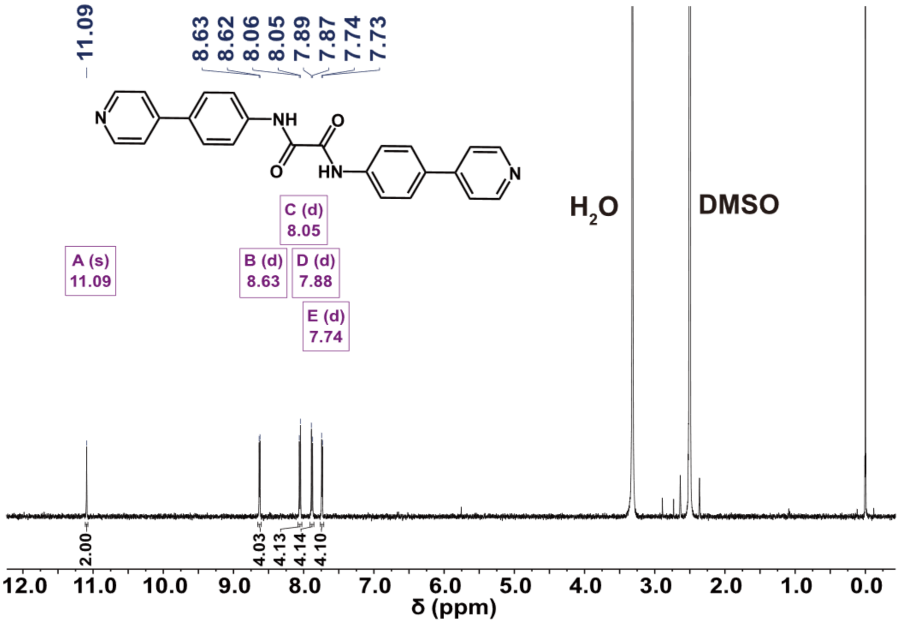

Figure S1. ^{1}H NMR spectrum of N^1,N^2-bis(4-(pyridin-4-yl)phenyl)oxalamide (500 MHz, 298.15 K, DMSO-d_6).

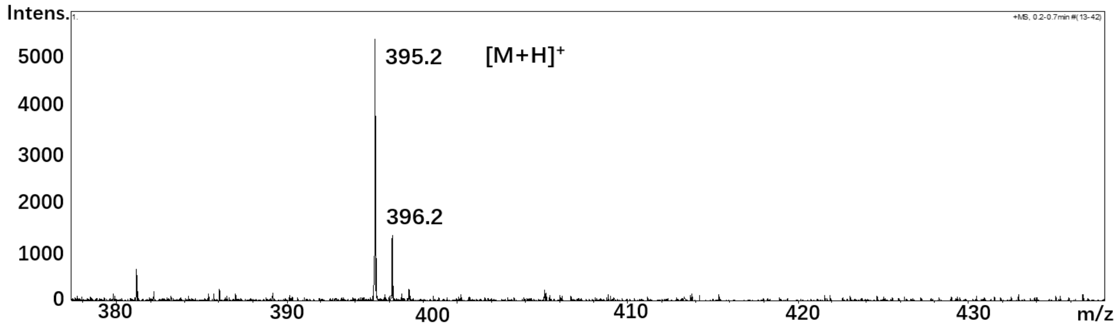

Figure S2. Mass spectrum of N[1],N[2]-bis(4-(pyridin-4-yl)phenyl)oxalamide.

BPP N[1],N[2]-Bis(4-(pyridin-4-yl)phenyl)oxalamide (100 mg, 0.25 mmol) and methyl iodide (2 mL, 0.03 mol) were dissolved in N,N-dimethylformamide (10 mL), and the mixture was heated at 120°C for 12 hours. After the reaction was complete, the solution was cooled to room temperature, and the resulting brown solid was collected by filtration. The solid was washed three times with dichloromethane (20 mL) and dried under vacuum, yielding the brown product **BPP** (0.16 g, 95%). ^{1}H NMR (400 MHz, DMSO-d_6) δ 11.30 (s, 2H), 8.98 (d, J = 6.4 Hz, 4H), 8.49 (d, J = 6.3 Hz, 4H), 8.17 (d, J = 2.9 Hz, 8H), 4.31 (s, 6H). ^{13}C NMR (126 MHz, DMSO) δ 158.63, 153.28, 145.39, 141.12, 129.01, 128.86, 123.23, 120.88, 46.85. LC-MS (m/z): calculated for $C_{26}H_{24}N_4O_2^+$ ([M - 2I]$^{2+}$): 212.1; found: 212.1.

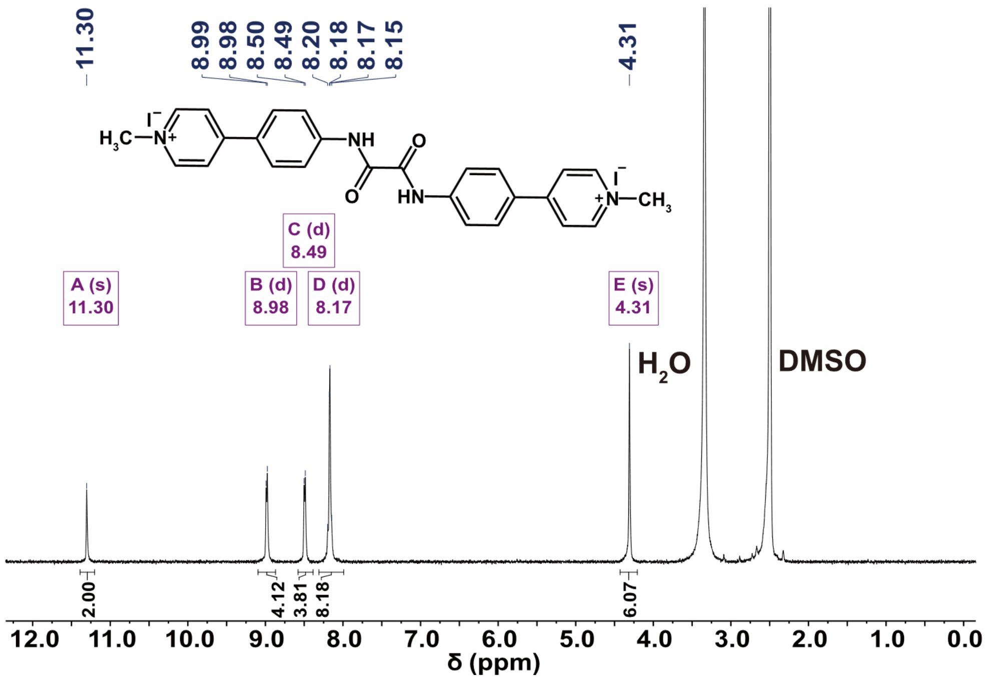

Figure S3. ^{1}H NMR spectrum of **BPP** (400 MHz, 298.15 K, DMSO-d_6).

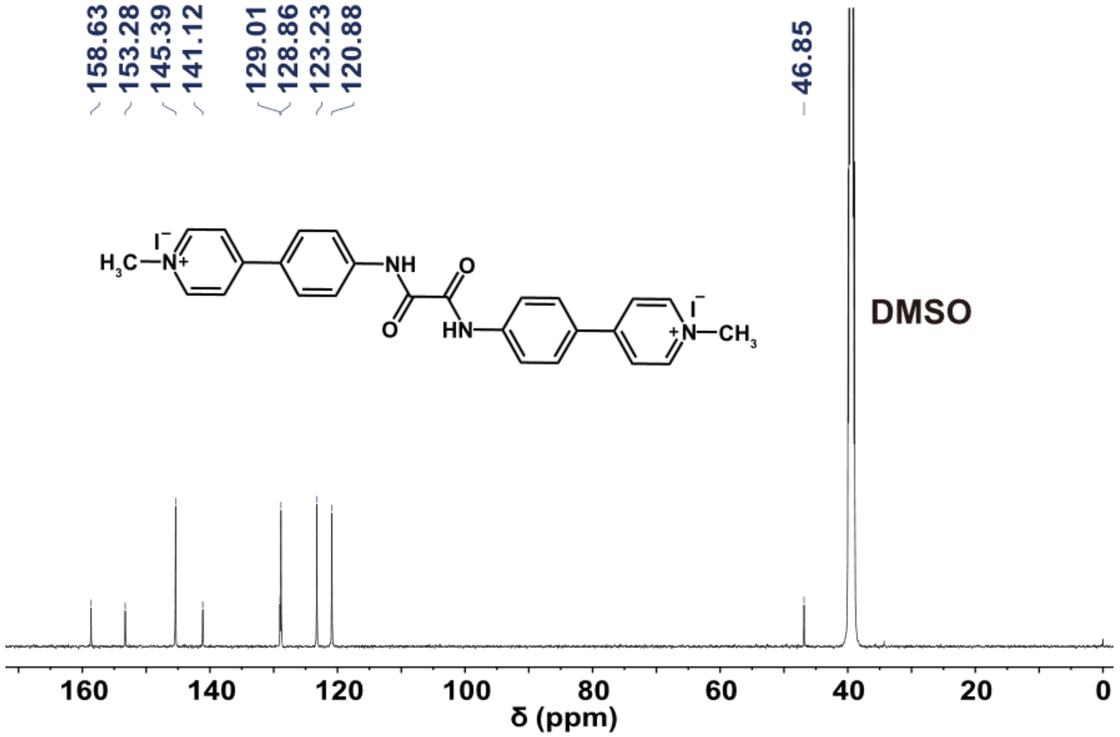

Figure S4. ^{13}C NMR spectrum of **BPP** (126 MHz, 298.15 K, DMSO-d_6).

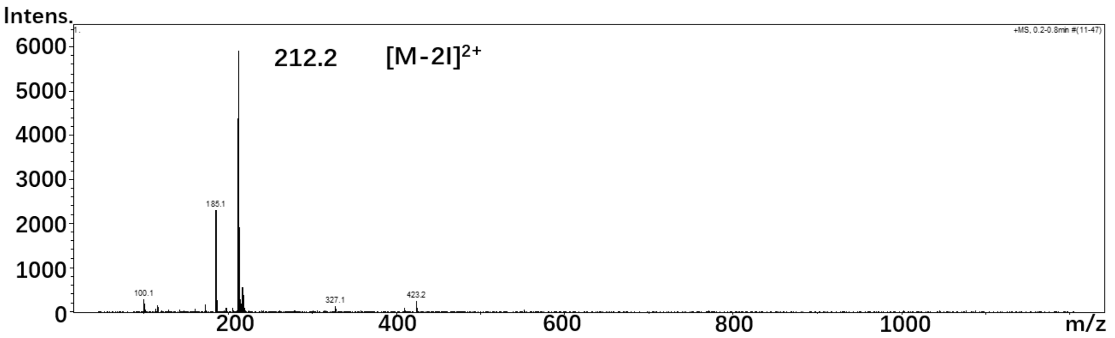

Figure S5. Mass spectrum of **BPP**.

3. NMR Spectra of BPP and its CB[8]-mediated complexes

The changes in chemical shifts (Δδ) of individual BPP protons upon complexation with CB[8] and Na^+ were analyzed and correlated with their positions along the molecular framework. Proton assignments were made based on COSY and NOESY spectra. The resulting Δδ profiles (**Figure S6**) provide a spatial representation of the average CB[8] location along the BPP axis and thereby reflect the relative mobility of the macrocycle within each assembly. Specifically, the Δδ distributions for BPP_2·$CB[8]_1$ (blue), BPP_2·$CB[8]_2$ (orange), and BPP_2·$CB[8]_2$·Na (green) correspond to distinct positional preferences of CB[8]: axial sliding across the central region, end-residing confinement, and ion-bridged contraction toward the center, respectively.

(a)

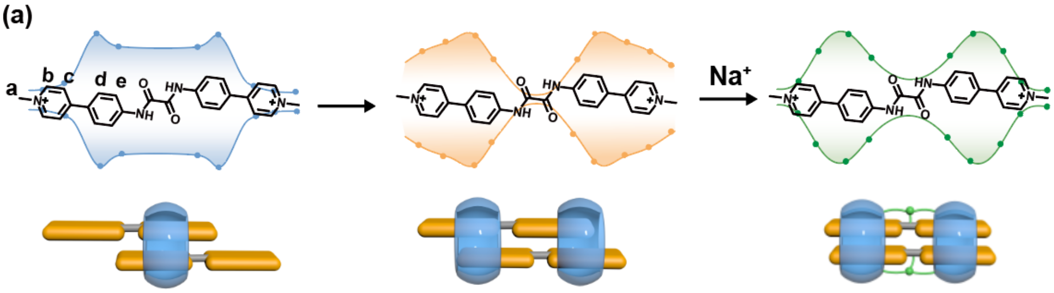

(b)

	Δδ (ppm)		
	BPP_2·$CB[8]_1$	BPP_2·$CB[8]_2$	BPP_2·$CB[8]_2$·Na
a	0.14	0.38	0.12
b	0.15	0.58	0.24
c	0.21	0.69	0.87
d	0.50	0.79	1.04
e	0.43	0.40	0.62

Figure S6. (a) Schematic illustration of CB[8] trajectory in the **BPP_2·$CB[8]_1$**(blue), **BPP_2·$CB[8]_2$**(orange), and **BPP_2·$CB[8]_2$·Na**(green); (b) Chemical shift changes (Δδ, ppm) of **BPP** protons upon complexation with CB[8] and Na^+ (NaBr) in D_2O at 298 K.

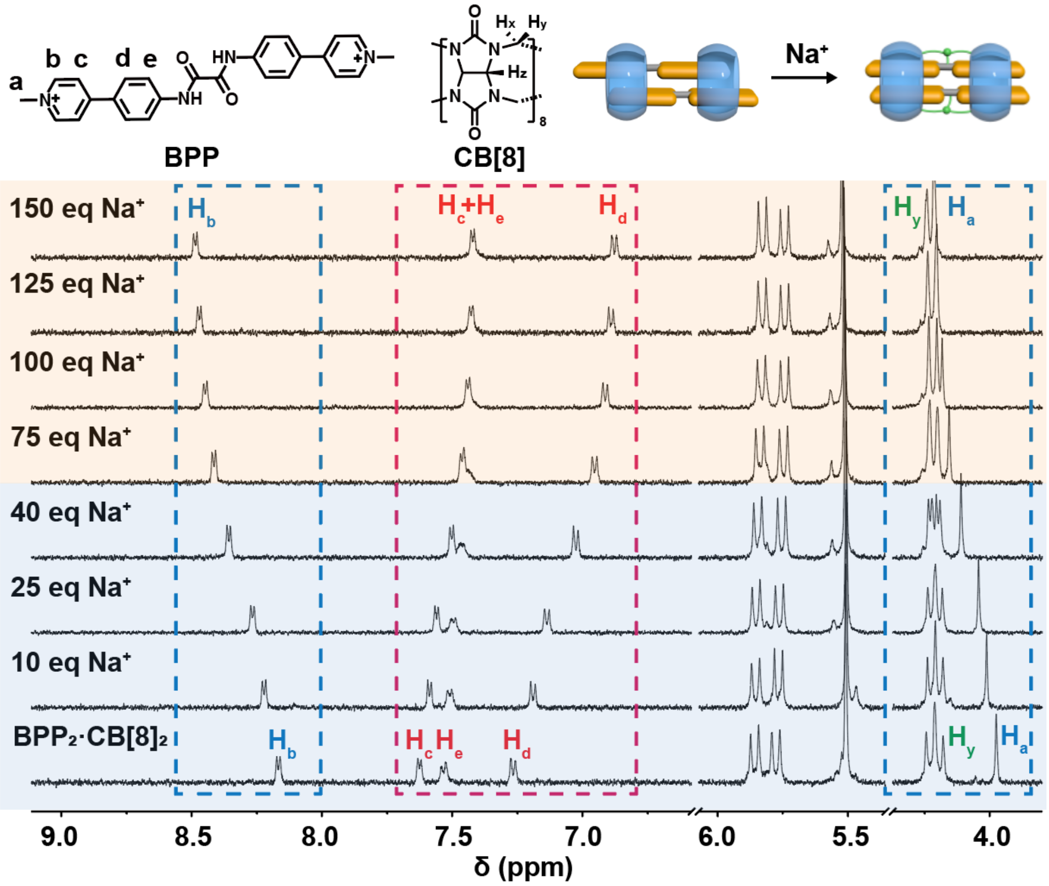

Figure S7. NMR titration spectra of $BPP_2 \cdot CB[8]_2$ upon addition of varying equivalents of NaBr (D_2O, 298 K).

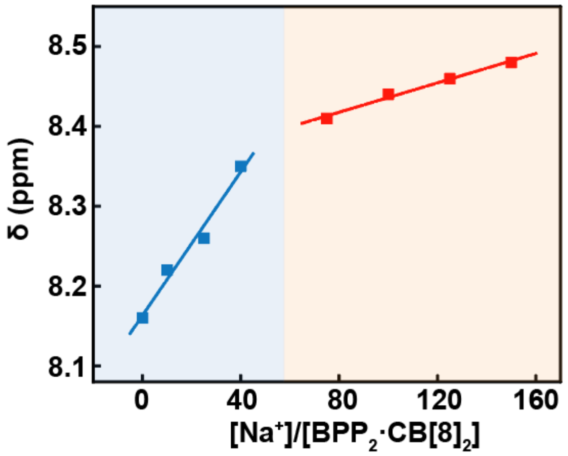

Figure S8. Variation of the H_b proton (see Figure S7) chemical shift in $BPP_2 \cdot CB[8]_2$ with increasing equivalents of sodium ions, exhibiting a distinct two-stage behavior.

The DOSY experiments were conducted on the complexes formed by BPP and CB[8]. The diffusion coefficient of the 2:2 complex was measured to be ~1.6×10^{-10} m^2/s, while that of the 2:1 complex was ~1.9×10^{-10} m^2/s.

Table S1. Diffusion coefficients of different assemblies of **BPP**.

Species	D ($\times10^{-10}$ m^2/s)
**$BPP_2\cdot CB[8]_1$**	**1.92**
**$BPP_2\cdot CB[8]_2$**	**1.59**
**$BPP_2\cdot CB[8]_2\cdot Na$**	**1.62**

The NOESY experiments were carried out using a modified version of the Bruker sequence noesygpph19, with the mixing time of 500 ms.

The COSY experiments were carried out using a modified version of the Bruker sequence cosydfgpph19.

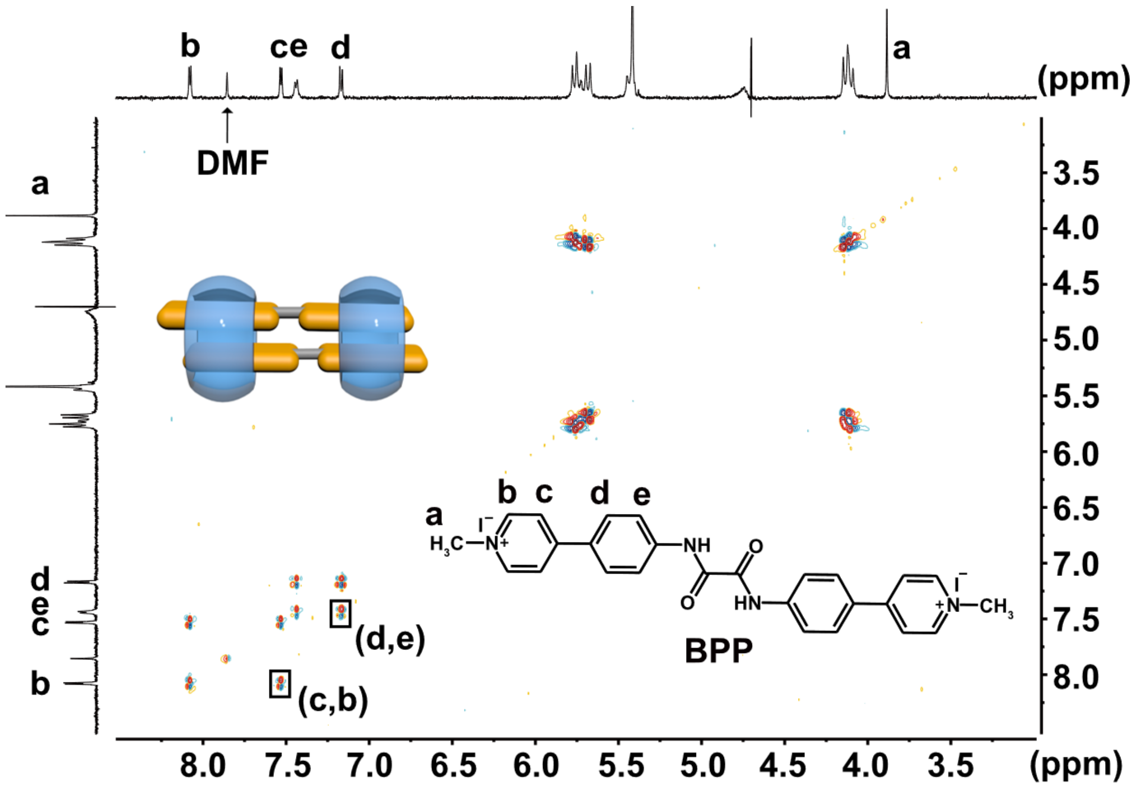

Figure S9. ^1H-^1H COSY spectrum (600 MHz, D_2O, 298.15 K) of **$BPP_2\cdot CB[8]_2$**. Key correlation peaks are labeled in the spectrum.

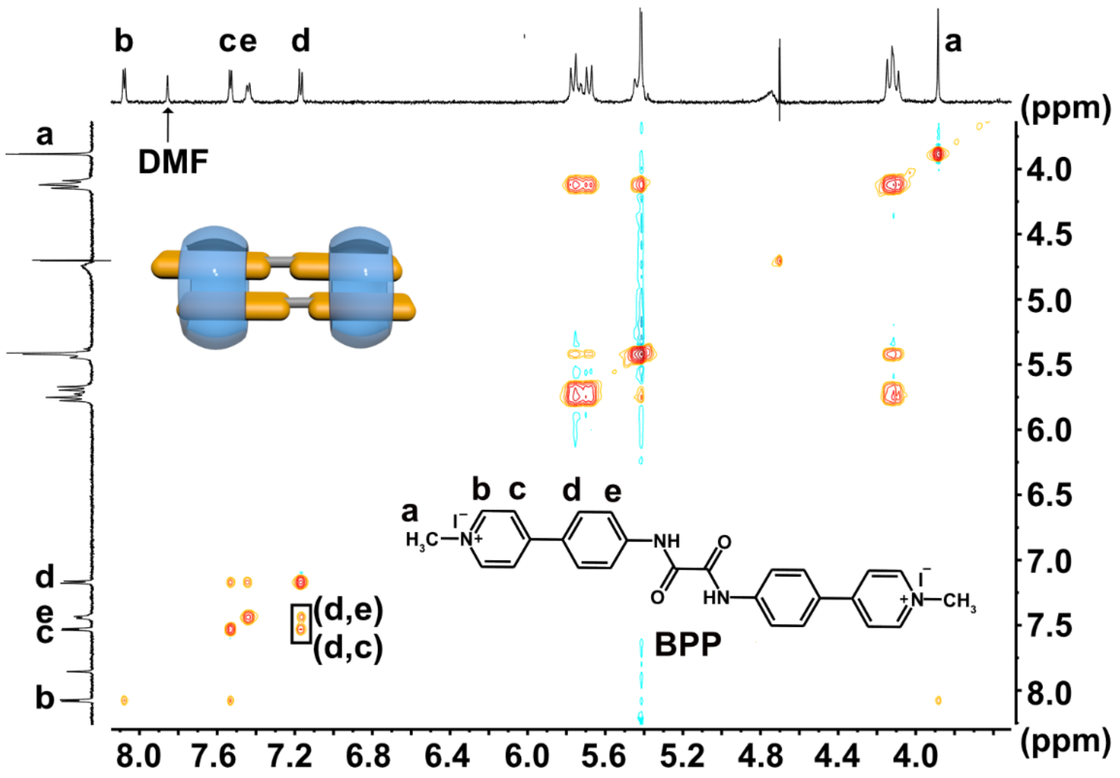

Figure S10. ^{1}H-^{1}H NOESY spectrum (600 MHz, D_2O, 298 K) of **BPP_2·CB[8]$_2$**. Key correlation peaks are labeled in the spectrum.

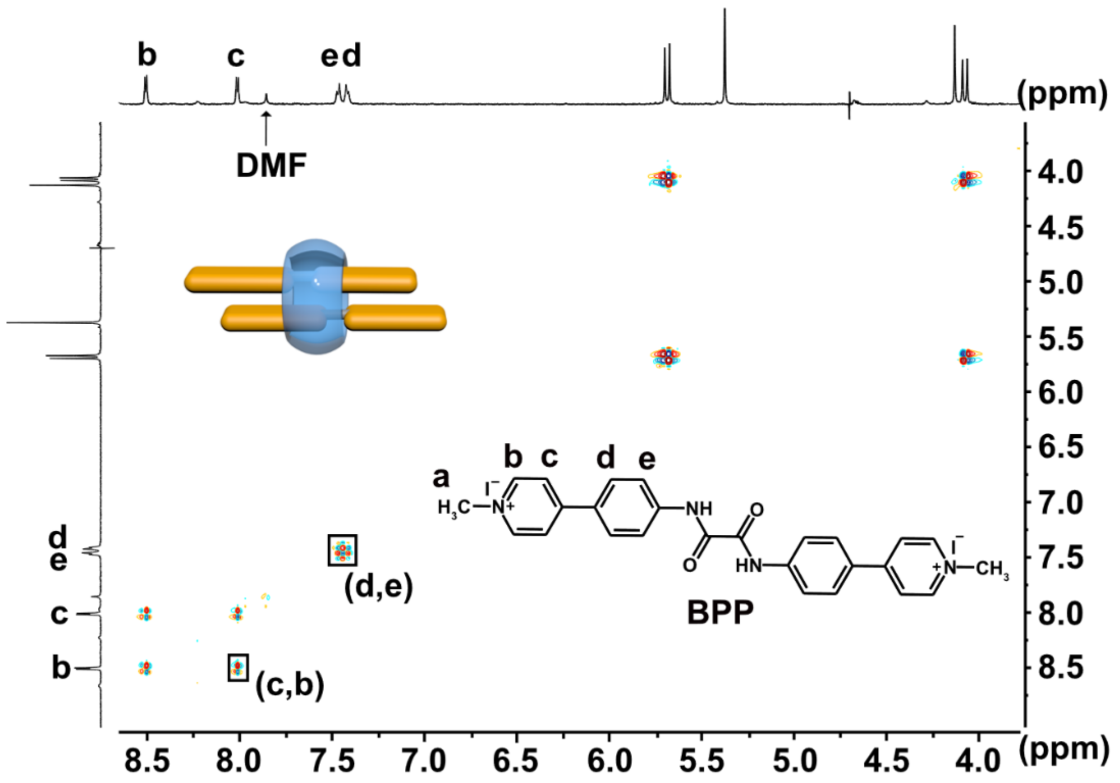

Figure S11. ^{1}H-^{1}H COSY spectrum (600 MHz, D_2O, 298.15 K) of **BPP_2·CB[8]$_1$**. Key correlation peaks are labeled in the spectrum.

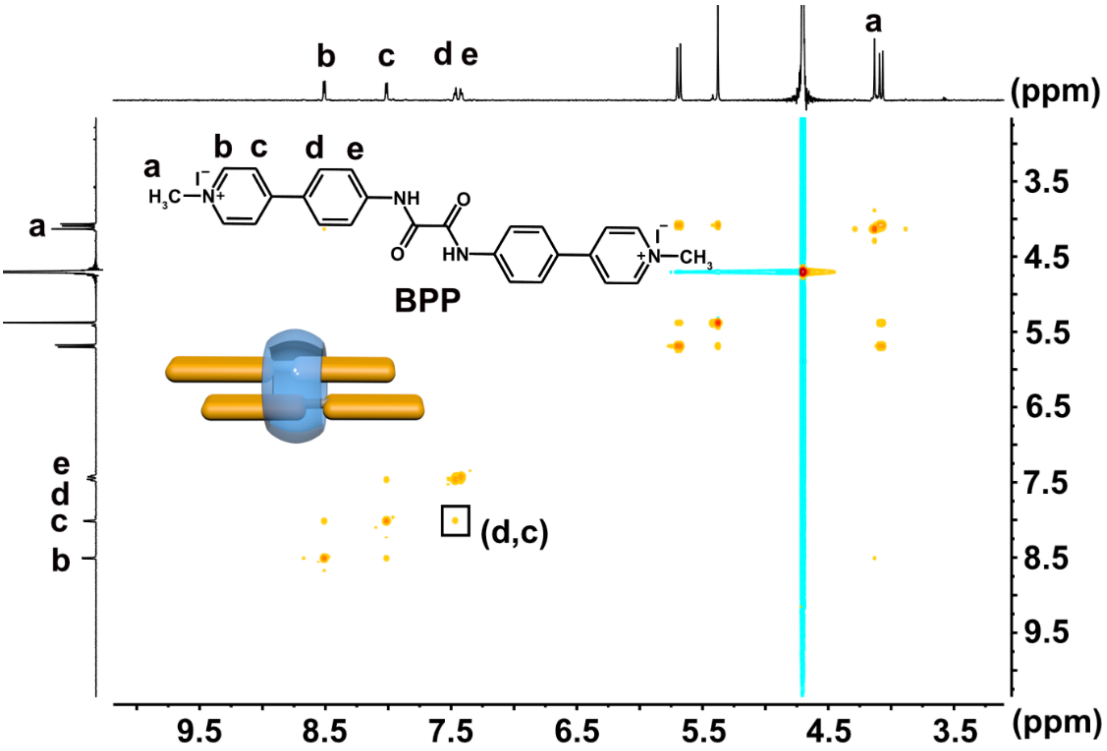

Figure S12. ^{1}H-^{1}H NOESY spectrum (600 MHz, D_2O, 298 K) of $\mathbf{BPP_2{\cdot}CB[8]_1}$. Key correlation peaks are labeled in the spectrum.

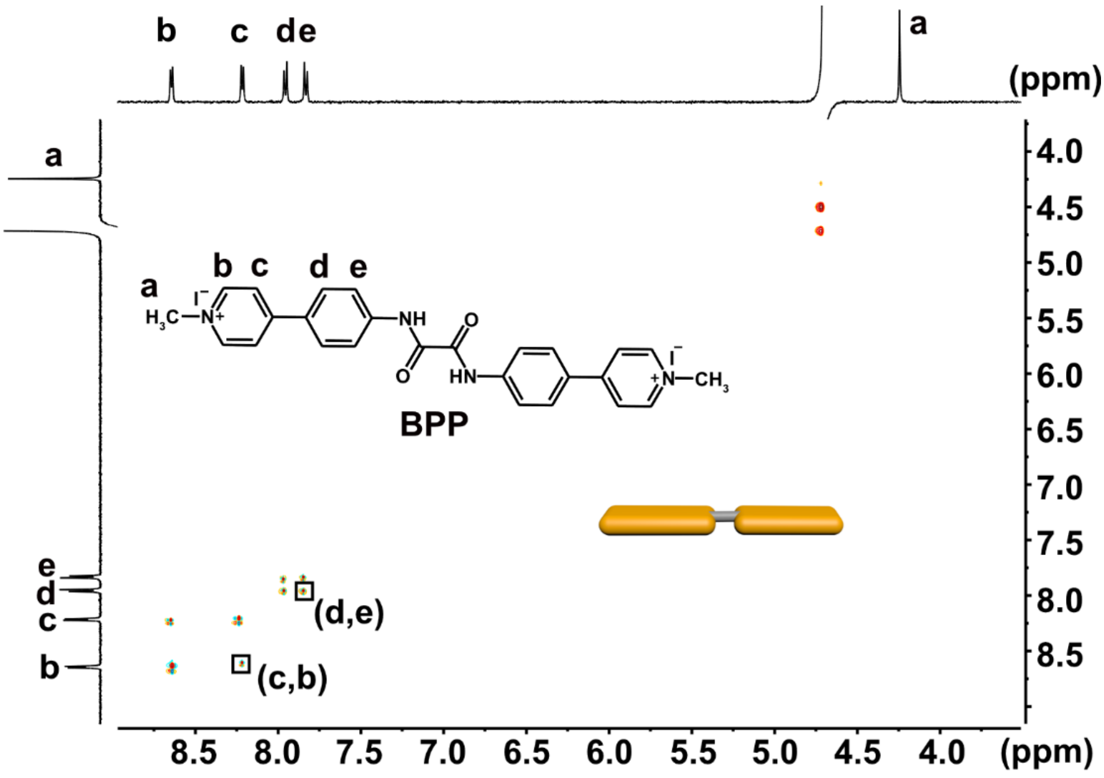

Figure S13. ^{1}H-^{1}H COSY spectrum (600 MHz, D_2O, 298.15 K) of **BPP**. Key correlation peaks are labeled in the spectrum.

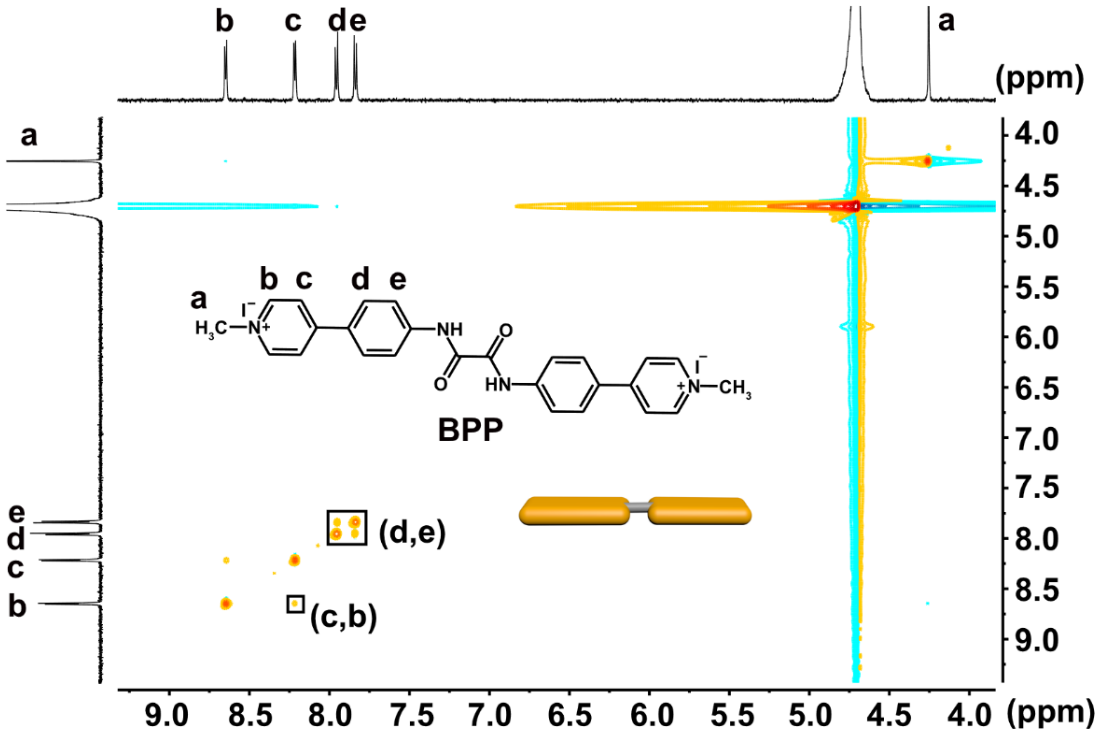

Figure S14. ^{1}H-^{1}H NOESY spectrum (600 MHz, D_2O, 298 K) of **BPP**. Key correlation peaks are labeled in the spectrum.

4. Photophysical study on BPP and its CB[8]-mediated complexes

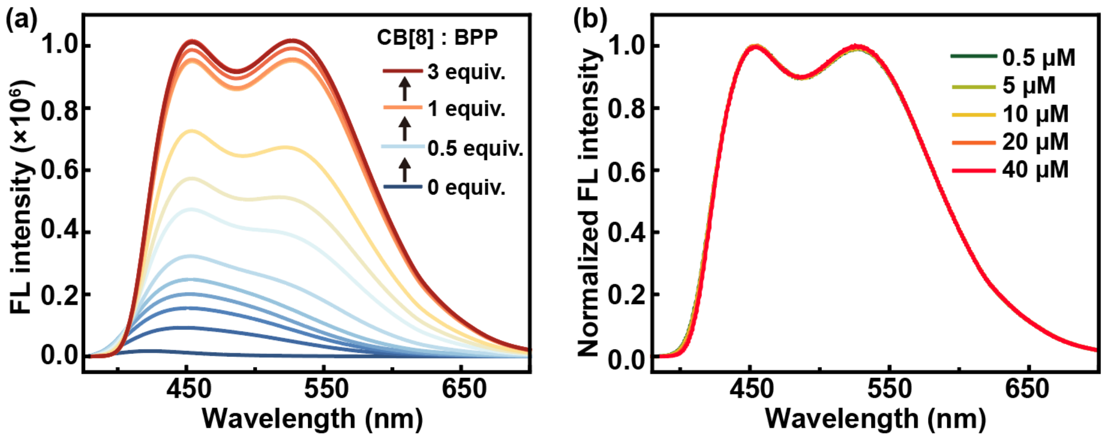

Figure S15. (a) Fluorescence spectra of **BPP** (40 μM) upon complexation with varying equivalents of CB[8]. (b) Normalized fluorescence spectra of **$BPP_2 \cdot CB[8]_2$** at different BPP concentrations (Ex = 350 nm).

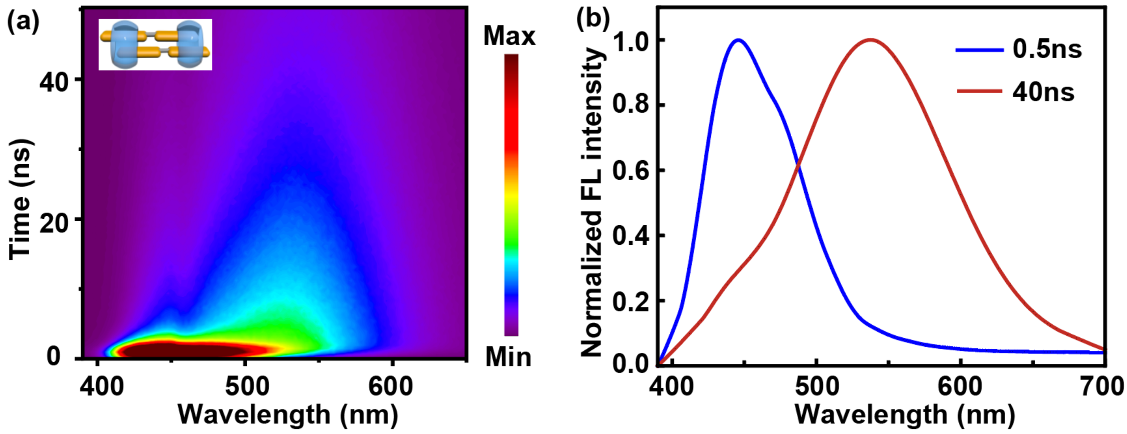

Figure S16. (a) Time-resolved fluorescence spectra of **$BPP_2 \cdot CB[8]_2$**. (b) Fluorescence spectra extracted from delay times of 0.5 ns and 40 ns, respectively. The sample is prepared with a BPP concentration of 20 μM at 298 K in water.

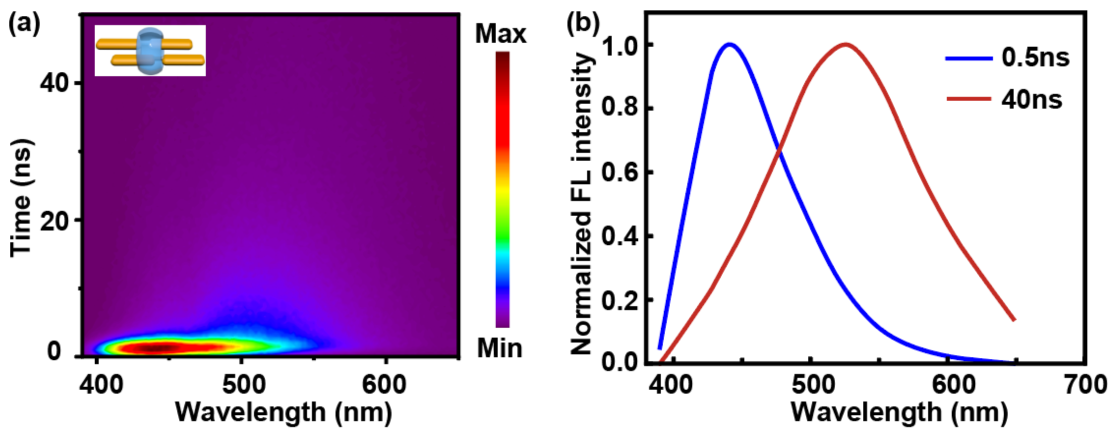

Figure S17. (a) Time-resolved fluorescence spectra of **BPP_2·CB[8]$_1$**. (b) Fluorescence spectra extracted from delay times of 0.5 ns and 40 ns, respectively. The sample is prepared with a BPP concentration of 20 μM at 298 K in water.

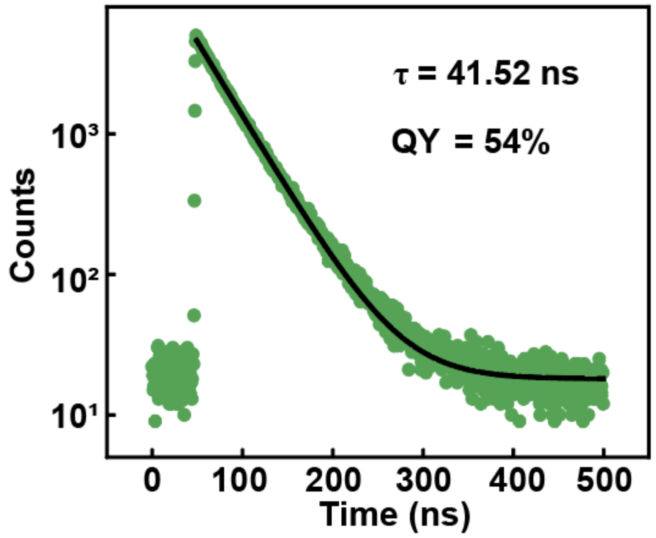

Figure S18. Fluorescence lifetime decay of BPP_2·CB[8]$_2$·Na (20 μM based on BPP units) monitored at 520 nm.

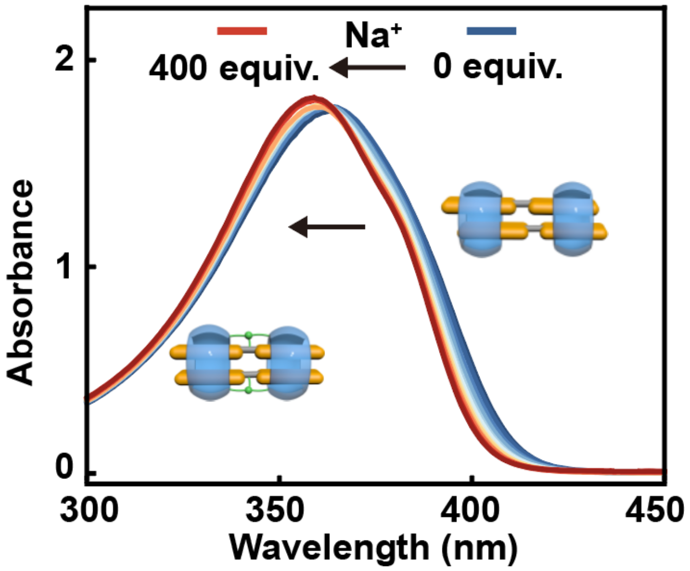

Figure S19. UV–vis absorption spectra of $\mathbf{BPP_2{\cdot}CB[8]_2}$ (40 μM based on BPP units) upon addition of varying equivalents of sodium ions (NaBr).

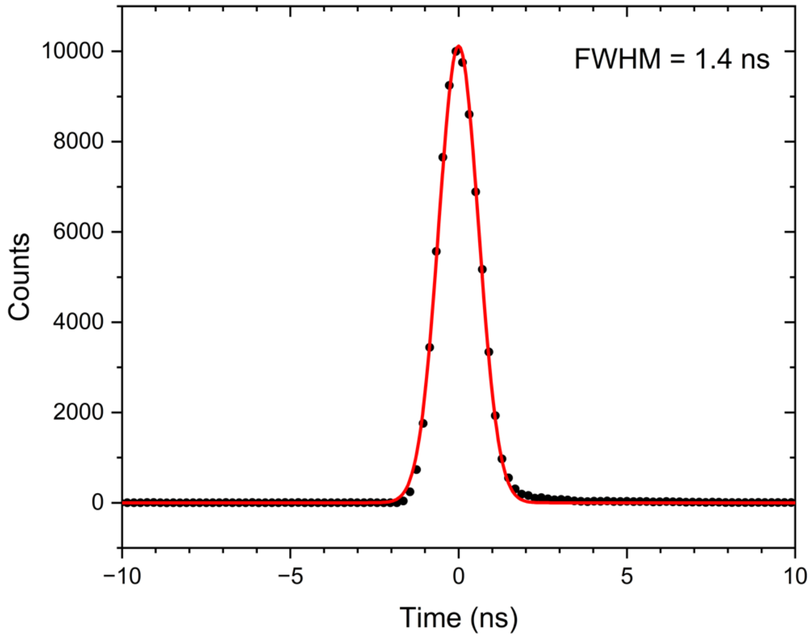

Figure S20. Instrument response function of TCSPC measurements.

5. Transient absorption of BPP and its CB[8]-mediated complexes

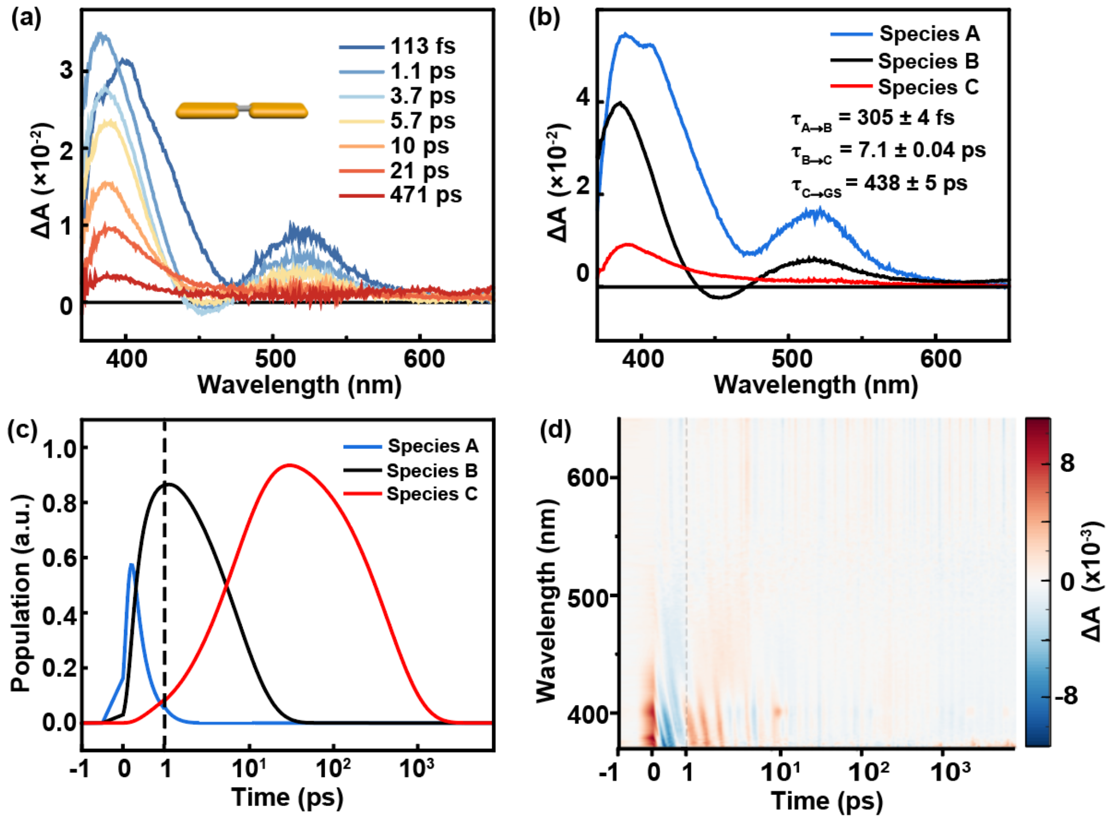

Figure S21. (a) TA spectra (UV-Vis) of **BPP** in H_2O (in air) at 298 K following excitation at 350 nm; (b) species associated spectra from fitting the raw data using the kinetic model A→B→C→GS; (c) model population kinetics; and (d) residual plot.

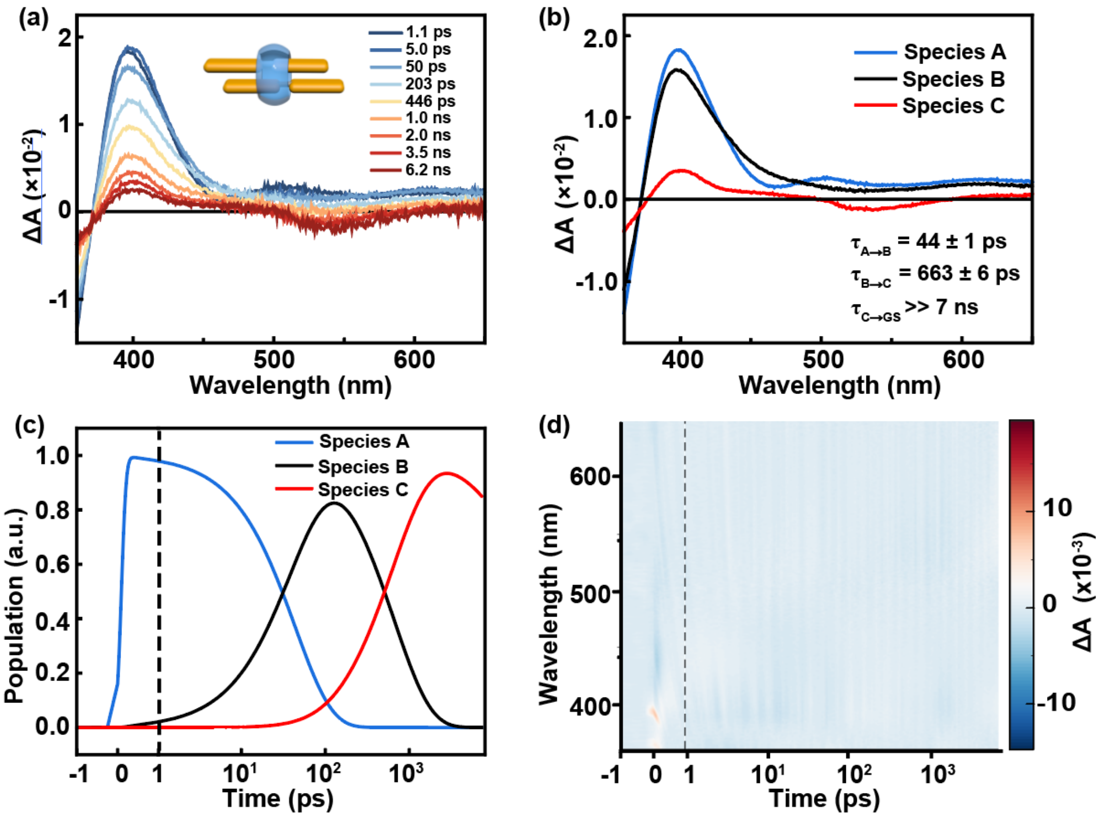

Figure S22. (a) TA spectra (UV-Vis) of **BPP$_2$·CB[8]$_1$** in H_2O (in air) at 298 K following excitation at 350 nm; (b) species associated spectra from fitting the raw data using the kinetic model A→B→C→GS; (c) model population kinetics; and (d) residual plot.

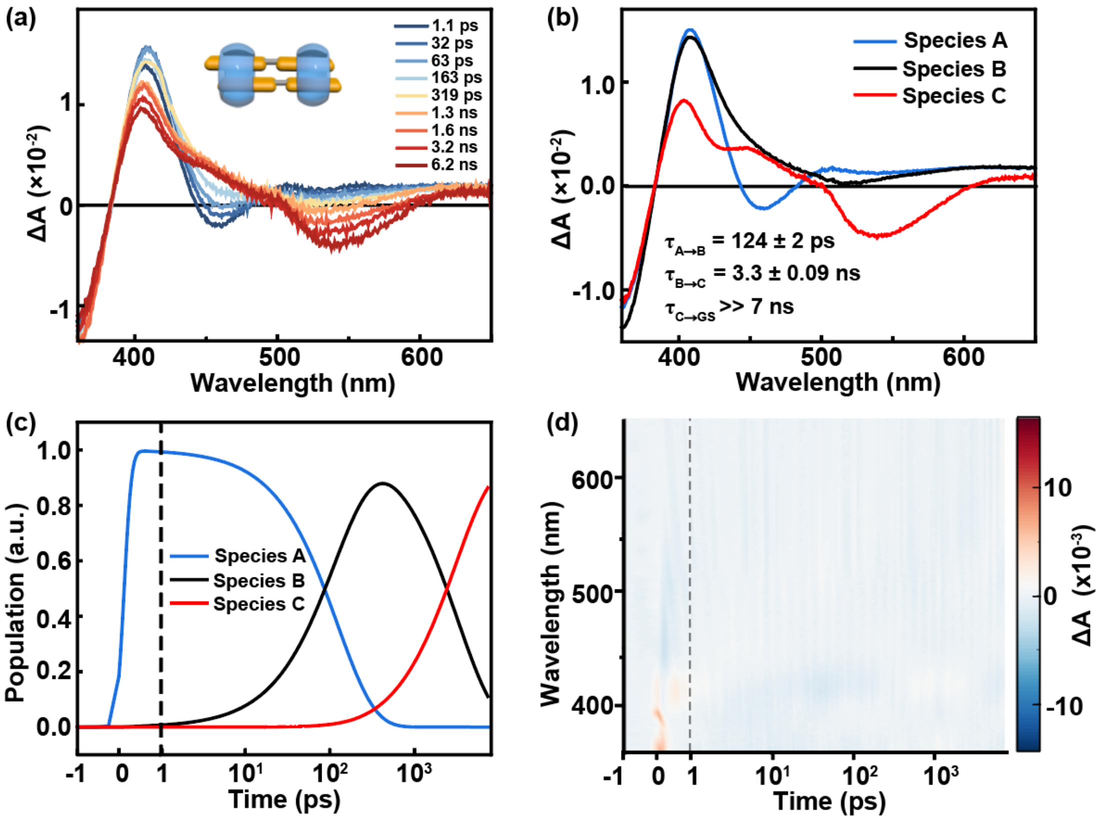

Figure S23. (a) TA spectra (UV-Vis) of **BPP_2·CB[8]$_2$** in H_2O (in air) at 298 K following excitation at 350 nm; (b) species associated spectra from fitting the raw data using the kinetic model A→B→C→GS; (c) model population kinetics; and (d) residual plot.

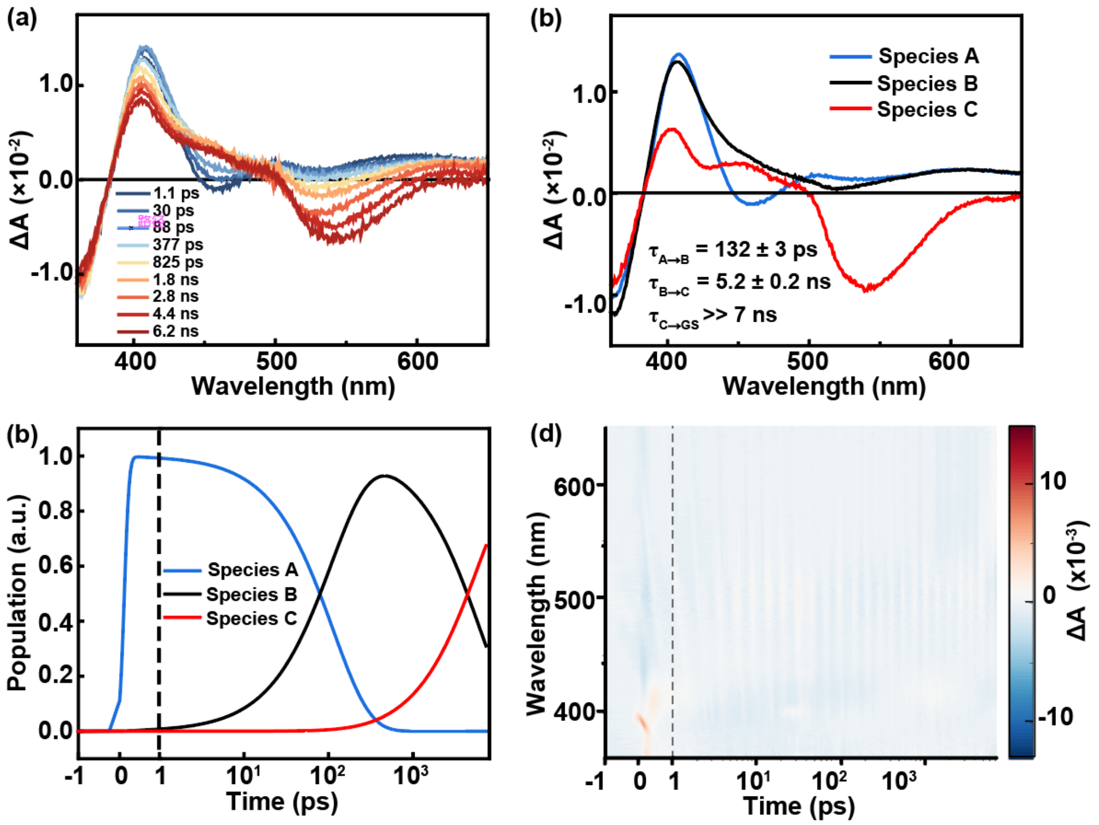

Figure S24. (a) TA spectra (UV-Vis) of **BPP$_2$·CB[8]$_2$·Na** (100 equiv. NaBr) in H_2O (in air) at 298 K following excitation at 350 nm; (b) species associated spectra from fitting the raw data using the kinetic model A→B→C→GS; (c) model population kinetics; and (d) residual plot.

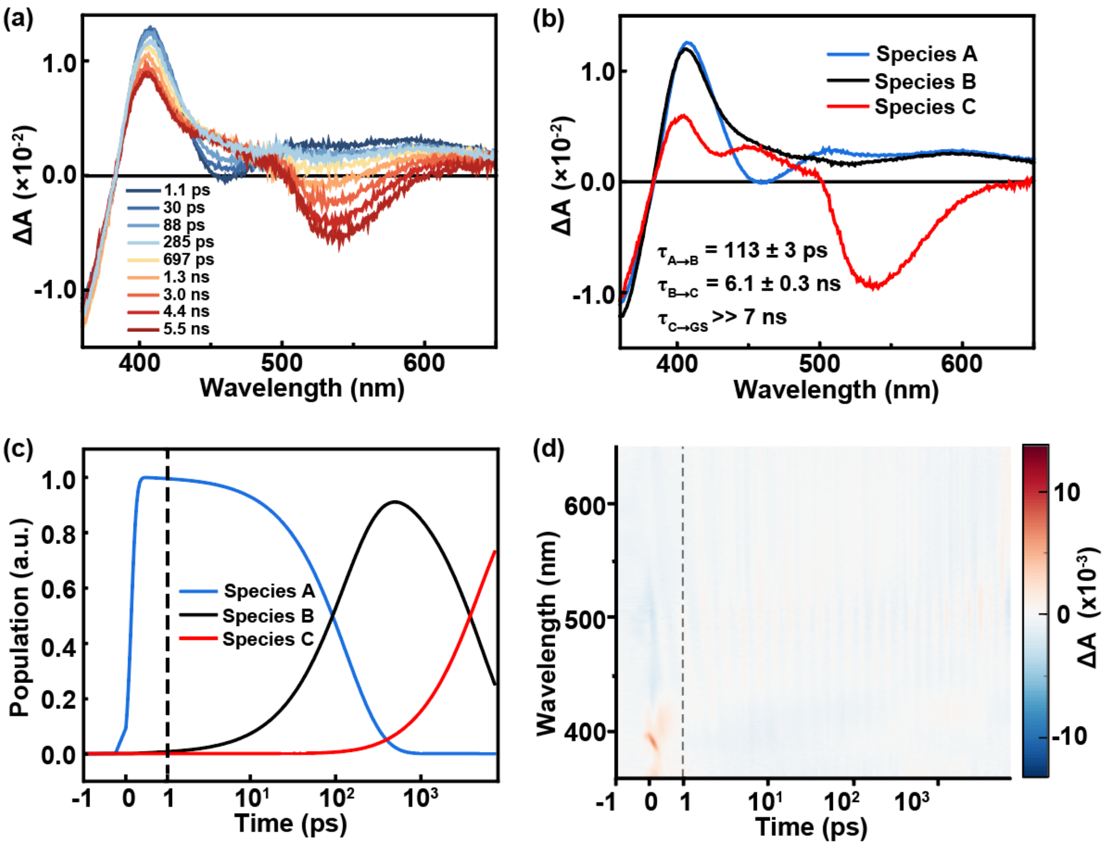

Figure S25. (a) TA spectra (UV-Vis) of **BPP_2·CB[8]$_2$·Na** (200 equiv. NaBr) in H_2O (in air) at 298 K following excitation at 350 nm; (b) species associated spectra from fitting the raw data using the kinetic model A→B→C→GS; (c) model population kinetics; and (d) residual plot.

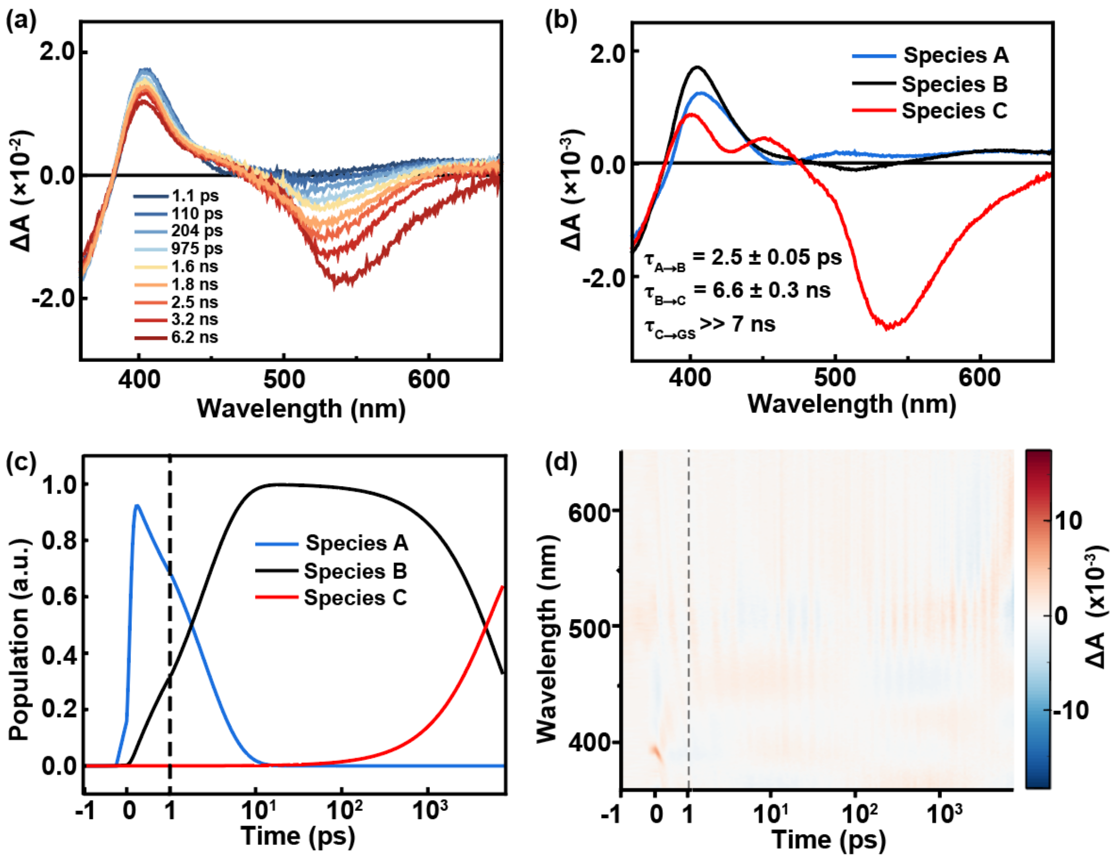

Figure S26. (a) TA spectra (UV-Vis) of **BPP$_2$·CB[8]$_2$·Na** (300 equiv. NaBr) in H_2O (in air) at 298 K following excitation at 350 nm; (b) species associated spectra from fitting the raw data using the kinetic model A→B→C→GS; (c) model population kinetics; and (d) residual plot.

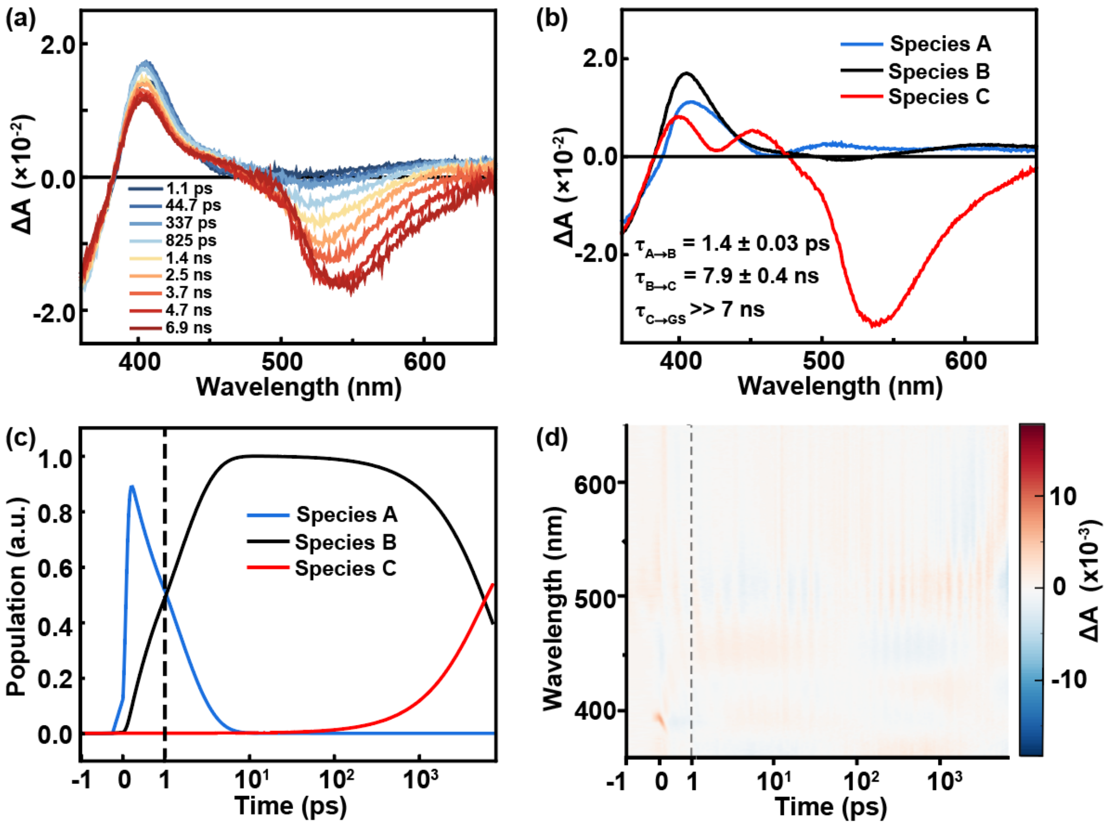

Figure S27. (a) TA spectra (UV-Vis) of **BPP$_2$·CB[8]$_2$·Na** (500 equiv. NaBr) in H_2O (in air) at 298 K following excitation at 350 nm; (b) species associated spectra from fitting the raw data using the kinetic model A→B→C→GS; (c) model population kinetics; and (d) residual plot.

6. Single crystal results

Single crystals were grown by adding appropriate amounts of NaBr to aqueous solutions of the free guest or the corresponding complex. The added salts increase the ionic strength of the solution, which helps reduce electrostatic repulsion and promote aggregation/crystallization. Crystals suitable for X-ray diffraction were obtained by slow evaporation of water at room temperature under ambient conditions.

Table S2. Crystal data and structure refinement for **$BPP_2 \cdot CB[8]_2 \cdot Na$**

Parameters	
Formula weight	6054.67
Temperature/K	273(2)
Crystal system	Triclinic
Space group	$P\bar{1}$
**a** (Å)	17.4310 (13)
**b** (Å)	18.1874 (13)
**c** (Å)	23.3337 (16)
**α** (°)	112.476
**β** (°)	90.959
**γ** (°)	115.016
Volume(Å^3)	6052.0 (8)
Z	1
Density (g/cm^3)	1.667
F(000)	3068
Radiation	0.71073
Goodness-of-fit on F^2	2.96
Final R indexes [I>=2σ (I)]	R_1 = 0.2347, wR_2 = 0.5812
Final R indexes [all data]	R_1 = 0.2491, wR_2 = 0.6002
CCDC	2417619

Table S3. Crystal data and structure refinement for **BPP**

Parameters	
Formula weight	631.30
Temperature/K	273(2)
Crystal system	Monoclinic
Space group	$P2_1$/n
**a** (Å)	6.6386(5)
**b** (Å)	11.0450(9)
**c** (Å)	17.4193(14)
**α** (°)	90.00
**β** (°)	95.658(2)
**γ** (°)	90.00
Volume($Å^3$)	1271.02(17)
Z	2
Density (g/cm^3)	1.650
F(000)	624
Radiation	0.71073
Goodness-of-fit on F^2	1.106
Final R indexes [I>=2σ (I)]	R_1 = 0.0812, wR_2 = 0.2225
Final R indexes [all data]	R_1 = 0.0822 wR_2 = 0.2250
CCDC	2444592

Comment on residual electron density and checkCIF alerts for the BPP monomer structure. The refined structure of the BPP monomer contains disordered iodine and bromine atoms, and the remaining checkCIF alerts are associated primarily with residual electron density near these heavy-atom sites. To clarify the origin and structural significance of these features, the analysis below is provided.

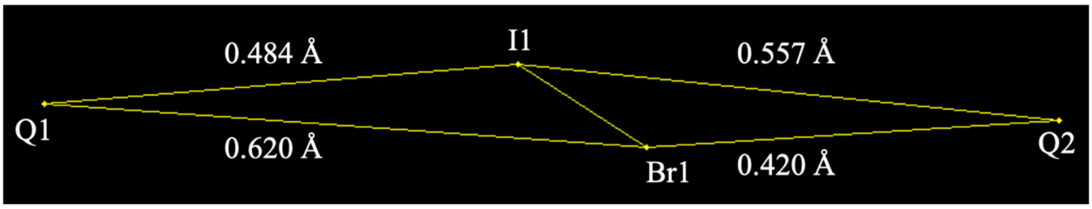

Figure S28. Distances between the major residual electron density peaks and the disordered heavy-atom sites in the BPP monomer crystal structure. The two largest positive peaks (Q1 and Q2) are located within 1 Å of the disordered I/Br positions, consistent with residual density arising from Fourier truncation artifacts around the heavy atoms.

The crystal structure contains disordered iodine (I) and bromine (Br) atoms, both of which are heavy elements whose electron density distributions exhibit characteristic Fourier truncation artifacts. As shown in **Figure S28**, the two largest positive residual peaks (Q1 and Q2) are located within 1 Å of the disordered I/Br sites. Given their proximity and magnitude, these peaks are attributable not to unmodeled atoms, but to incompleteness in the Fourier synthesis arising from the strong, localized electron density of the heavy atoms. Likewise, the most prominent negative peaks are also located within 1 Å of the same heavy-atom positions, further supporting their origin as truncation-induced ripples in the residual electron density map. This behavior stems from the substantial atomic number contrast between the heavy halogens (I, Br) and the lighter atoms (C, H, N, and O) comprising the organic cation, which results in pronounced density gradients and the corresponding checkCIF alerts. Despite these local residual features, the positional and orientational parameters of the organic cation remain well defined and chemically reasonable; accordingly, the core structural motif, including the cation conformation, hydrogen-bonding network, and overall packing arrangement, is not affected.

7. Geometry optimization

Initial structures were pre-optimized using the GFN2-xTB[2] method with optimization level of extreme, using the analytical linearized Poisson-Boltzmann (ALPB) [3] solvation model of water in xTB 6.5.1[4]. Geometry optimizations were subsequently performed at the B97-3c[5] level of theory using the conductor-like polarizable continuum model (CPCM) [6,7] of water. Then single point (SP) energies were calculated at the ωB97M-V[8]/def2-TZVP[9] level with a strong SCF convergence criterion, employing the solvation model based on density (SMD) [10] of water. RIJCOSX[11,12] was used to accelerate calculations and geometrical counterpoise (gCP)[13] was applied to empirically correct basis set superposition error(BSSE). All DFT calculations were carried out using ORCA 6.1.0-f.0[14]. All optimized molecular structures were rendered by VMD 1.9.3[15].

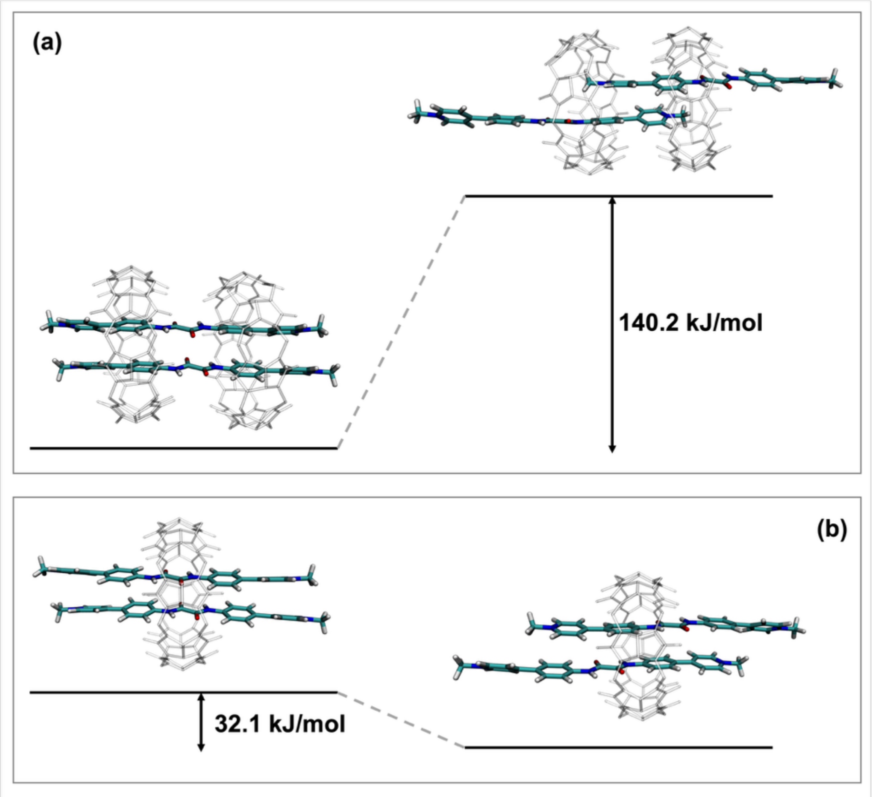

Figure S29. DFT-optimized structures of representative slipped conformations of (a) **BPP_2·CB[8]$_2$** and (b) **BPP_2·CB[8]$_1$**, together with their relative energies.

8. References

[1] Day, A., Arnold, A. P., Blanch, R. J., Snushall, B. "Controlling Factors in the Synthesis of Cucurbituril and Its Homologues" *J. Org. Chem.* **2001**, *66* (24), 8094-8100.

[2] Bannwarth, C.; Ehlert, S.; Grimme, S. GFN2-xTB-An Accurate and Broadly Parametrized Self-Consistent Tight-Binding Quantum Chemical Method with Multipole Electrostatics and Density-Dependent Dispersion Contributions. *J Chem Theory Comput* **2019**, *15* (3), 1652–1671.

[3] Ehlert, S.; Stahn, M.; Spicher, S.; Grimme, S. Robust and Efficient Implicit Solvation Model for Fast Semiempirical Methods. *J Chem Theory Comput* **2021**, *17* (7), 4250–4261.

[4] Bannwarth, C.; Caldeweyher, E.; Ehlert, S.; Hansen, A.; Pracht, P.; Seibert, J.; Spicher, S.; Grimme, S. Extended tight-binding quantum chemistry methods. *WIREs Computational Molecular Science* **2020**, *11* (2), e1493.

[5] Brandenburg, J. G.; Bannwarth, C.; Hansen, A.; Grimme, S. B97-3c: A revised low-cost variant of the B97-D density functional method. *J Chem Phys* **2018**, *148* (6), 064104.

[6] Barone, V.; Cossi, M. Quantum Calculation of Molecular Energies and Energy Gradients in Solution by a Conductor Solvent Model. *The Journal of Physical Chemistry A* **1998**, *102* (11), 1995–2001.

[7] Garcia-Rates, M.; Neese, F. Effect of the Solute Cavity on the Solvation Energy and its Derivatives within the Framework of the Gaussian Charge Scheme. *J Comput Chem* **2020**, *41* (9), 922–939.

[8] Mardirossian, N.; Head-Gordon, M. ωB97M-V: A combinatorially optimized, range-separated hybrid, meta-GGA density functional with VV10 nonlocal correlation. *J Chem Phys* **2016**, *144* (21), 214110.

[9] Weigend, F.; Ahlrichs, R. Balanced basis sets of split valence, triple zeta valence and quadruple zeta valence quality for H to Rn: Design and assessment of accuracy. *Phys Chem Chem Phys* **2005**, *7* (18), 3297–3305.

[10] Marenich, A. V.; Cramer, C. J.; Truhlar, D. G. Universal Solvation Model Based on Solute Electron Density and on a Continuum Model of the Solvent Defined by the Bulk Dielectric Constant and Atomic Surface Tensions. *The Journal of Physical Chemistry B* **2009**, *113* (18), 6378–6396.

[11] Neese, F.; Wennmohs, F.; Hansen, A.; Becker, U. Efficient, approximate and parallel Hartree–Fock and hybrid DFT calculations. A 'chain-of-spheres' algorithm for the Hartree–Fock exchange. *Chemical Physics* **2009**, *356* (1-3), 98–109.

[12] Helmich-Paris, B.; de Souza, B.; Neese, F.; Izsak, R. An improved chain of spheres for exchange algorithm. *J Chem Phys* **2021**, *155* (10), 104109.

[13] Kruse, H.; Grimme, S. A geometrical correction for the inter- and intra-molecular basis set superposition error in Hartree-Fock and density functional theory calculations for large systems. *J Chem Phys* **2012**, *136* (15), 154101.

[14] Neese, F. Software Update: The ORCA Program System—Version 6.0. *WIREs Computational Molecular Science* **2025**, *15* (2), e70019.

[15] Humphrey, W.; Dalke, A.; Schulten, K. VMD: Visual molecular dynamics. *Journal of Molecular Graphics* **1996**, *14* (1), 33–38.
